# Potential Implications of Interactions between Fe and S on Cereal Fe Biofortification

**DOI:** 10.3390/ijms21082827

**Published:** 2020-04-18

**Authors:** Yuta Kawakami, Navreet K. Bhullar

**Affiliations:** Plant Biotechnology, Department of Biology, ETH Zurich, Universitätstrasse 2, 8092 Zurich, Switzerland; yuta.kawakami@biol.ethz.ch

**Keywords:** Fe and S interaction, Fe biofortification, cereals, phytosiderophores, nicotianamine, Strategy II, Strategy I

## Abstract

Iron (Fe) and sulfur (S) are two essential elements for plants, whose interrelation is indispensable for numerous physiological processes. In particular, Fe homeostasis in cereal species is profoundly connected to S nutrition because phytosiderophores, which are the metal chelators required for Fe uptake and translocation in cereals, are derived from a S-containing amino acid, methionine. To date, various biotechnological cereal Fe biofortification strategies involving modulation of genes underlying Fe homeostasis have been reported. Meanwhile, the resultant Fe-biofortified crops have been minimally characterized from the perspective of interaction between Fe and S, in spite of the significance of the crosstalk between the two elements in cereals. Here, we intend to highlight the relevance of Fe and S interrelation in cereal Fe homeostasis and illustrate the potential implications it has to offer for future cereal Fe biofortification studies.

## 1. Introduction

Iron (Fe) is an essential element for living organisms. Being a metal element with variable oxidization states, Fe mediates a range of redox reactions, including electron transport in mitochondria or oxygen transport by red blood cells in humans and mammals. Despite its critical biochemical functions, human dietary Fe intake is often found to be suboptimal, posing a significant health risk to people worldwide [1]. Anemia, the most common outcome of human Fe deficiency, is estimated to affect a quarter of the global population [1]. In developing regions, where anemia is particularly prevalent [2], the occurrence of insufficient Fe intake is commonly associated with poverty—owing to economic disparities and food distribution challenges, the diets are predominated by one or few foods, such as starch-rich cereals. Cereals are relatively inexpensive and rich in calories, but poor in bioavailable Fe, as well as other nutrients [3].

As a countermeasure against Fe deficiency, intrinsic improvement of Fe content as well as bioavailability in the cereal grains, known as cereal biofortification, is considered to be an effective and sustainable strategy [4]. To date, various biotechnological cereal biofortification strategies involving enhancement of Fe uptake, (re)translocation, and storage mechanisms have been reported [5,6,7,8,9]. However, although much attention has been paid to the biotechnological strategies and the resultant increment in grain Fe concentration, only a limited extent of characterization has been made on other physiological or agro-morphological aspects of Fe-biofortified crops [10,11].

In this review, we aim to propose potentially intriguing yet untapped aspects of Fe-biofortified cereal crops in light of interaction between Fe and sulfur (S) in plants. We first illustrate the mechanisms underlying Fe homeostasis in cereals, highlighting its commonality and diversity among graminaceous species, as well as the latest insights. Secondly, we summarize the currently reported strategies for cereal Fe biofortification, mainly in rice (*Oryza sativa*) but also in other cereal crops. Subsequently, we elaborate on the uptake, transport, metabolism, and biological functions of S in cereals, with a focus on its roles related to the homeostasis of Fe and other metal species. Finally, we cover the spheres of crosstalk between Fe and S in cereals crops that could offer particular implications for future Fe biofortification efforts, namely, (1) effect of crop S status on Fe acquisition and translocation, (2) hydrogen sulfide as a potential regulator of crop Fe homeostasis, (3) Fe as a buffer for sulfide damage in the rice rhizosphere, and (4) roles of S-containing biomolecules in response to concomitant increase in heavy metal uptake by Fe-biofortified crops.

## 2. Fe Uptake and Transport in Cereals

In this section, we provide a concise overview of the mechanisms underlying Fe homeostasis in cereal crops, highlighting their diversity among cereal species and recent insights. Readers interested in a detailed review on the mechanisms, especially with an extensive graphical illustration, are referred to preceding articles [9,12,13,14].

### 2.1. Fe Uptake System and Phytosiderophore Synthesis

In aerobic soil conditions, Fe is mostly present as sparingly soluble ferric oxides, which cannot be taken up by plants as such. So as to take up sufficient Fe from the soil, graminaceous species employ a mode of Fe acquisition called Strategy II [15,16]. In Strategy-II Fe uptake, plants secrete a group of Fe(III) chelators called mugineic-acid family phytosiderophores (PS) from the roots via TRANSPORTER OF MUGENEIC ACID 1 (TOM1) [17]. The resultant Fe(III)–PS complex are taken up by the cereal plants through YELLOW STRIPE 1 (YS1) or YS1-LIKE (YSL) transporters, such as maize (*Zea mays*) YS1 (ZmYS1), barley (*Hordeum vulgare*) YS1 (HvYS1), or rice YSL15 (OsYSL15) [18,19,20,21].

**Figure 1:**
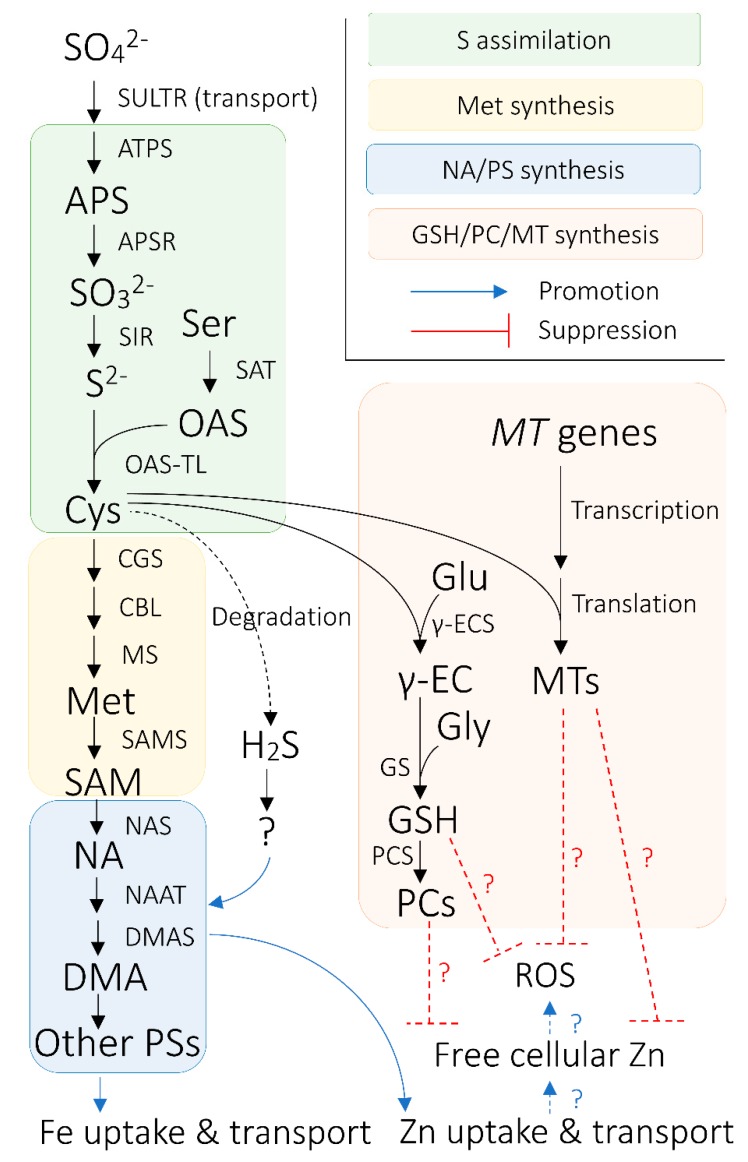
Overview of the connection between sulfur (S) nutrition and iron (Fe) nutrition in cereals plants. Black arrows indicate enzymatic synthetic reactions, unless otherwise indicated. Blue arrows signify promotional effects between biological processes, whereas red bars with flat ends indicate suppressive effects between biological agents and processes. Arrows and bars with dotted lines indicate relationships between biological processes/agents that are hypothetically pronounced in Fe-biofortified crops. Hydrogen sulfide (H_2_S) is hypothesized to regulate nicotianamine (NA)/phytosiderophores (PS) synthesis via unknown biological mechanisms or agents. Abbreviations are as follows: APS: ADENOSINE PHOSPHOSULFATE; APSR: APS REDUCTASE; ATPS: ATP SULFURYLASE; CBL: CYSTATHIONINE β-LYASE; CGS: CYSTATHIONINE γ-SYNTHASE; Cys: cysteine; DMA: deoxymugineic acid; DMAS: DMA SYNTHASE; Glu: glutamate; Gly: glycine; GS: GLUTATHIONE SYNTHETASE; GSH: glutathione; Met: methionine; MS: Met synthase; MT: METALLOTHIONEIN; NAAT: NA AMINOTRANSFERASE; NAS: NA SYNTHASE; OAS: *O*-acetylserine; OAS-TL: OAS (THIOL) LYASE; PC; phytochelatin; PCS: PC synthase; ROS: reactive oxygen species; S^2−^: sulfide; SAM: *S*-adenosyl methionine; SAMS: SAM SYNTHASE; Ser: serine; SO_3_^2−^: sulfite; SO_4_^2−^: sulfate; SULTR: SULFATE TRANSPORTER; Zn: zinc; γ-EC: glutamylcysteine; γ-ECS: γ-EC SYNTHETASE.

PS are produced from *S*-adenosyl methionine (SAM; AdoMet), which originates from the methionine cycle through a sequence of enzymatic reactions [22,23,24] (Figure 1), putatively in a intracellular vesicle derived from rough endoplasmic reticulum (rER) [25,26,27,28,29,30]. Firstly, nicotianamine (NA) is synthesized via trimerization of SAM by NA SYNTHASE (NAS) [31]. Then, deoxymugineic acid (DMA) is synthesized from NA in the reactions mediated by NA AMINOTRANSFERASE (NAAT) and DMA SYNTHASE (DMAS) [32,33] (Figure 1). DMA is the only type of PS produced by rice, wheat (*Triticum aestivum*), and maize [34,35], whereas barley and some other graminaceous species can further convert DMA into other members of PS, some of which have stronger Fe(III)-binding capability than DMA [36,37,38,39]. The amount of PS secreted from roots also varies among graminaceous species, positively correlating with the crops’ tolerance to Fe deficiency—barley is the most tolerant crop with the largest PS secretion, whereas rice is the least tolerant with the smallest PS secretion [40].

Relatively modest level of PS secretion from rice may be associated with the environment to which rice and its relatives have adapted [41,42]. Rice is commonly cultivated in water-logged paddy fields, where anaerobic soil conditions prevail. In deoxygenated soil, Fe is reduced to soluble Fe(II) and is fairly available for plant uptake [43]. Thus, in contrast to other cereal species [44], rice partially adopts Strategy-I Fe uptake, in which Fe(II) ions are directly taken up by IRON-REGULATED TRANSPORTERs (IRTs) [41,45]. Although a functional Strategy-II uptake system is present in rice, when Fe(II) ions are abundant in the rhizosphere, Fe acquisition is mainly achieved through Strategy-I uptake, and genes involved in Strategy-II Fe uptake are down-regulated [43,46].

### 2.2. Potential Diversity in the Manner of Fe Uptake and Radial Fe Transport in Roots

Among cereal species, there are also variations in root anatomy that possibly affect the site of Fe uptake in roots, as well as the fashion of subsequent within-root radial Fe transport required for xylem Fe loading. 

The number of Casparian strips, which block apoplastic flow of water and nutrients across the root cell layers, is one of such anatomical variations. Almost all vascular plants have endodermis, a cell layer with a Casparian strip surrounding the root stele [47]. Moreover, the majority of the plants investigated possess exodermis, another cell layer with a Casparian strip beneath the epidermis [48]. Although rice and maize are commonly found to possess both endodermal and exodermal Casparian strips (exodermal species), wheat and barley tend to be void of exodermis (non-exodermal species), though these characteristics may vary depending on the type of roots, genotype, and stress conditions [48,49,50,51,52]. In exodermal species, nutrients should be taken up either by epidermal or exodermal cells because they are the only cell layers contacting the soil solution [47]. In non-exodermal species, on the other hand, cortical and endodermal cells can contribute to the uptake due to the absence of a barrier for the apoplastic flow of nutrients into the cortex from the external environment [47]. In line with this notion, the expression of Fe(III)-PS uptake transporter in maize, ZmYS1, is confined to epidermal cells [53]. Meanwhile, HvYS1 expression in barley can be found not only in epidermal cells but also in the cortex [18]. Furthermore, a Fe(III)-DMA transporter HvYSL2 is expressed in root endodermal cells [54]. These observations collectively suggest a potential variation in Fe uptake site in roots among different cereal species.

Subsequent to uptake from the rhizosphere, Fe should be radially transported to the root stele for xylem loading. The manner of such within-root Fe transport may also differ, especially between rice and other cereal species, owing to the difference in root anatomy. As a species well adapted to flooded conditions, rice has a highly developed aerenchyma between the endodermis and exodermis, through which air is supplied for the root cells [55]. Because only a small number of cells are alive in rice aerenchyma, there is little symplastic connection between the rice epi/exodermis and stele [56]. This distinct root internal structure in rice requires the nutrients to be apoplastically transported across the cortex before entering the stele, which is putatively mediated by a pair of efflux and influx transporters at the exodermis and endodermis, respectively [57,58]. Rice Fe transporters involved in this transport are currently unknown, but the fact that OsYSL15 is induced not only in epidermis but also in cortex upon Fe deficiency [21,59], even though rice is an exodermal species, suggests the potential involvement of OsYSL15 in radial Fe transport in roots.

### 2.3. Fe Mobilization for Long-Distance and Intercellular Transport

**Figure 2:**
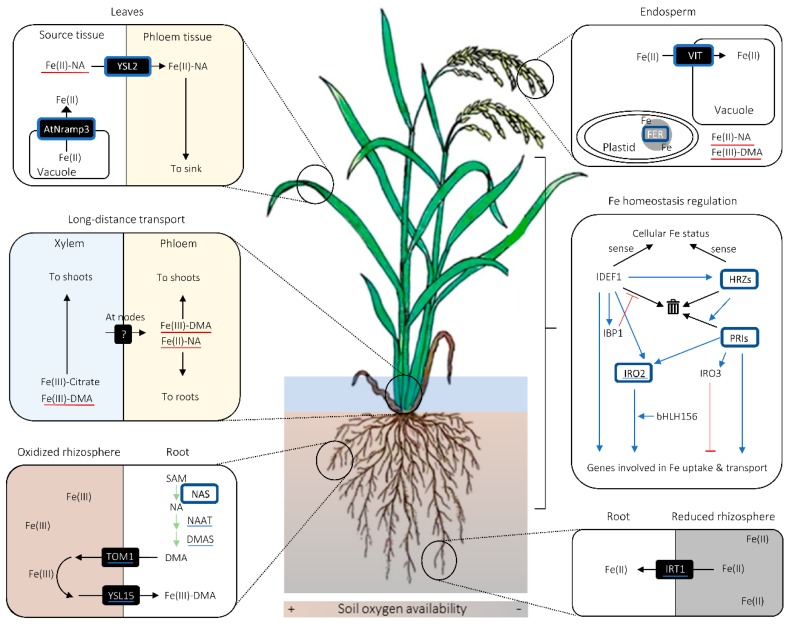
Graphical summary of important cereal iron (Fe) biofortification strategies. Proteins surrounded with blue squares are those whose modulation leads to increase in grain Fe concentration. Blue underlines indicate the molecules whose genes are up-regulated in rice lines overaccumulating nicotianamine (NA) or overexpressing *NA SYNTHASE* (*NAS*). Red underscores refer to the complexes whose amount is putatively increased in *NAS*-overexpressing lines. Black arrows indicate the movement of molecules unless otherwise noted, whereas green arrows signify synthetic enzymatic reactions. In the inset for Fe homeostasis regulation, blue arrows and red bars with flat ends indicate promotional and suppressive effects between molecular agents and/or processes, respectively. In the same inset, black arrows pointing to the bin indicate proteasomic degradation of proteins. Abbreviations are as follows: AtNRAMP3: NATURAL RESISTANCE-ASSOCIATED MACROPHAGE PROTEIN 3 from *Arabidopsis thaliana*; bHLH156: BASIC HELIX-LOOP-HELIX 156; DMA: deoxymugineic acid; DMAS: DMA SYNTHASE; FER: FERRITIN; HRZ: HEMERYTHRIN MOTIF-CONTAINING REALLY INTERESTING NEW GENE- AND ZINC-FINGER PROTEIN; IBP1: IDEF1-BINDING PROTEIN 1; IDEF1: IRON DEFICIENCY-RESPONSIVE ELEMENT-BINDING FACTOR 1; IRO2/3: IRON-RELATED TRANSCRIPTION FACTOR 2/3; IRT1: IRON-REGULATED TRANSPORTER 1; PRI: POSITIVE REGULATOR OF IRON DEFICIENCY RESPONSE; TOM1: TRANSPORTER OF MUGENEIC ACID 1; VIT: VACUOLAR IRON TRANSPORTER; YSL2/15: YELLOW STRIPE-1 LIKE 2/15.

Owing to its high redox activity and low solubility, Fe has to be chelated by organic ligands for its solubilization and transport in plants [60]. The main chelators for Fe long-distance transport in cereals are considered to be citrate, NA, and PS [61,62,63,64,65,66,67,68]. In xylem Fe transport, citrate seems to be the major chelator [61,62,64,65], whereas DMA plays a supplementary role, especially under Fe deficiency (Figure 2) [62,63]. As citrate is considered to be loaded onto xylem via FERRIC REDUCTASE DEFECTIVE LIKE 1 (FRDL1) transporter in a form unbound to Fe, there can be a transporter that mediates unchelated Fe loading onto xylem, which is yet to be identified [65]. The loading of DMA and NA onto xylem may be undertaken by TOM2 and EFFLUX TRANSPORTER OF NA 1 (ENA1), respectively [69,70].

In phloem, NA and/or DMA are likely to be the primary chelators for Fe (Figure 2) [61,66,71]. A Fe(II)-NA transporter YSL2, and possibly a Fe(III)-DMA transporter YSL18, contribute to Fe transport via phloem in rice [71,72,73], which suggests that Fe is primarily loaded onto phloem in a chelated form. It remains to be examined whether or not there is a phloem loading of chelator-free Fe.

Fe transferred from roots to shoots is once accumulated in stem nodes and distributed to various aboveground tissues, probably via phloem [74]. This implies that there is a mechanism mediating xylem-to-phloem Fe transfer in stem nodes (Figure 2), an organ that plays a central role in intervascular nutrient transfer and nutrient delivery to various aboveground tissues in graminaceous species [75,76]. The transporters mediating this putative xylem-to-phloem Fe transfer at stem nodes are yet to be identified. Besides being a hub for nutrient distribution, stem nodes pool nutrients in their apoplastic regions [77,78,79]. The role of citrate and citrate transporter FRDL1 in remobilizing Fe deposited in the apoplastic regions of the nodes has been described [80]. 

Graminaceous plants can also accumulate Fe in apoplastic spaces in roots, which can serve as a reservoir for Fe, especially under Fe-limiting conditions [81,82,83,84,85]. Phenolic compounds can contribute to solubilizing Fe precipitated in the root apoplastic regions. In rice, PHENOLICS EFFLUX ZERO1 (PEZ1) is identified as a transporter responsible for mobilizing precipitated Fe in the root xylem by pumping in protocatechuic acid (PCA) in the xylem sap [84]. In addition, PEZ2 provides phenolics to solubilize Fe in the apoplasm in the roots [85].

Route for Fe loading onto the grains may differ among cereal species as well. In wheat and barley, xylem is discontinued towards the grain [86,87]. On the other hand, there is no discontinuity of xylem towards developing rice grains [88,89]. This implies that the relative contribution of phloem-mediated source-to-sink Fe translocation to grain Fe concentration may differ among cereals.

In cereal species including rice, there is no symplastic connection between the maternal tissue and the filial tissues in grains [86,90]. Therefore, Fe must be transported though a pair of unknown efflux and influx transporters in order to be loaded onto the grain. After grain loading, Fe tends to accumulate at a higher concentration in the aleurone layer than in the endosperm [91,92]. In aleurone layer, Fe is often associated with phytic acids, which makes Fe unavailable for humans [91,93]. Fe transfer between endosperm and embryo seems to be mediated by YSL9 transporter in rice [94].

### 2.4. Fe Intracellular Homeostasis

Given the reactive nature of Fe, surplus Fe has to be sequestered in vacuole in plant cells [95]. On the other hand, organelles such as mitochondria and chloroplasts should be supplied with ample Fe to fulfill their physiological roles involving many redox reactions [96,97]. To meet these somewhat dilemmatic needs, Fe transport and storage in these organelles are tightly controlled. In the last decade, roles of transporters and Fe-binding agents in the regulation of intracellular Fe homeostasis have been gradually revealed in cereals, especially in rice.

MITOCHONDRIAL IRON TRANSPORTER (MIT) assumes a critical role in providing Fe for mitochondria [98]. As Fe importers for chloroplasts, Fe DEFICIENCY-RELATED 3 (FDR3) and FDR4 transporters were identified in maize [99,100]. Fe storage in plastids, particularly under sufficient or excess Fe conditions, is likely to be mediated by Fe storage protein FERRITIN (FER), whose genes encode transit peptides for plastid localization at its N-terminus [101,102,103]. In addition to FER, VACUOLAR IRON TRANSPORTERs (VITs) on the tonoplast promote compartmentalization of surplus Fe into vacuoles [104,105]. In rice, knockdown of a tonoplast-localized DMA influx transporter VACUOLAR MUGINEIC ACID TRANSPORTER (VMT) leads to higher root cell sap Fe concentration as well as lower xylem Fe concentration [106]. This implies that Fe once sequestered in vacuoles is stored as Fe(III) and can be exported again as Fe(III)-DMA. Moreover, it has been postulated that FERRIC REDUCTASE OXIDASE 1 (FRO1) localized on rice tonoplast can contribute to increasing the Fe availability for cytoplasm by reducing Fe(III) to Fe(II) in vacuoles [107]. Therefore, there may also be an unknown Fe(II) export mechanism from the vacuoles, in addition to the Fe(III)-DMA export machinery.

### 2.5. Fe Homeostasis Regulation

Genes involved in Fe uptake are up-regulated in response to Fe deficiency in graminaceous species, whereas they are down-regulated under Fe sufficiency [43,108,109,110,111]. An elaborate network of regulatory factors underlying such an adaptive response has been delineated recently in rice, which may be transferrable to other cereal crops. The factors that assume a pivotal role in the regulatory network in rice are IRON DEFICIENCY-RESPONSIVE ELEMENT-BINDING FACTOR 1 (IDEF1) and HEMERYTHRIN MOTIF-CONTAINING REALLY INTERESTING NEW GENE- AND ZINC-FINGER PROTEINs (HRZs), which are classified into Fe sensors by virtue of their putative capacity to alter their function to regulate Fe homeostasis by directly sensing the Fe availability in the cells [112]. 

IDEF1 is a positive regulator of Fe uptake-related genes, which can sense the Fe availability in cells by binding to Fe and other metal ions (Figure 2) [113,114,115]. IDEF1 interacts with a *cis*-element IDE1 in the promoter regions of Fe deficiency-responsive genes to up-regulate them [113,116]. As there is a diversity in amino acid sequence in the metal-binding region of IDEF1 among different graminaceous species, there may also be an inter-species functional diversity for IDEF1 [115]. *IDEF1* gene is expressed irrespective of the Fe status of the plant, but its protein is prone to 26S proteasome-dependent degradation under Fe-sufficient conditions (Figure 2) [117,118]. In Fe-deficient conditions, its degradation is prevented by IDEF1-BINDING PROTEINs (IBPs), and as a result, genes involved in Fe deficiency response are induced (Figure 2) [117]. *IBP* genes have many IDE1 motifs in their promoter regions and are positively regulated by IDEF1, thereby constituting a positive feedback loop between IDEF1 and IBP1 for Fe deficiency response (Figure 2) [117]. In rice, IDEF1 is known to positively regulate another transcription factor governing Fe deficiency response, called IRON-RELATED TRANSCRIPTION FACTOR 2 (IRO2) (Figure 2) [113,119]. IRO2 can induce genes involved in Strategy-II Fe uptake upon Fe deficiency by binding to their promoter regions [120]. It has been recently revealed that IRO2 requires another transcription factor BASIC HELIX-LOOP-HELIX 156 (bHLH156) to localize in the nucleus and regulate gene expression (Figure 2) [121].

In contrast to IDEF1, HRZ1 and HRZ2 are identified as negative regulators of Strategy-II Fe uptake in rice, especially when there is sufficient external Fe available [122]. Supposedly, HRZs sense Fe availability also by binding with Fe and other metal ions (Figure 2) [122]. Like IDEF1, HRZs are as well susceptible to 26S proteasome-dependent degradation in roots, but contrary to IDEF1, they are so regardless of the Fe status (Figure 2) [122]. HRZs negatively regulate Fe uptake and translocation by contributing to the proteasomic degradation of POSITIVE REGULATORS OF IRON HOMEOSTASIS (PRIs) through their ubiquitination (Figure 2) [123,124]. PRIs positively regulate the expression of *IRO2* and also *YSL2*, which is not regulated by IRO2 [124,125].

Interestingly, *HRZ*s are positively regulated by IDEF1 (Figure 2) [122]. Moreover, PRIs, which are deemed overall as positive regulators of Fe uptake and translocation, induce the expression of *IRO3*, which codes for a negative transcription factor for Fe deficiency response in rice (Figure 2) [123,124,126]. These interrelations between antagonistic regulatory factors could be part of a sophisticated machinery to prevent excessive Fe deficiency response.

There are several other characterized regulatory factors involved in Fe homeostasis regulation in rice, whose association with the abovementioned regulation network, with IDEF1 and HRZs at its core, is absent or unknown. In parallel to IDE1 and IDEF1, a pair of *cis*-element and *trans*-factor, namely, IDE2 and IDEF2, were found to positively regulate Fe deficiency response [116,127]. Moreover, rice bHLH133 is a transcription factor that contributes to Fe retention in roots under Fe-deficient conditions [128]. Rice homologues of IRON MANs (IMAs), which are regulatory factors identified in Arabidopsis (*Arabidopsis thaliana)* as a positive regulator of systemic Fe deficiency signaling, are also likely to have similar roles in rice [129].

One rare exception of a transcription factor related to Fe transport identified first in wheat is NO APICAL MERISTEM B-1 (NAM B-1) [130]. Wheat NAM B-1 promotes the senescence of vegetative tissues, which leads to increased translocation of Fe from leaves to grains along with other nutrients [130]. Although NAM B-1 homologue is identified in rice, it does not have an identical role as the wheat counterpart [131].

In addition to transcription factors, signal transduction through phytohormones such as auxin or jasmonates are suggested as being important components of Fe deficiency response [132,133]. NA is also speculated as being a possible Fe deficiency signaling molecule [29,112,134], given that the genes involved in Fe acquisition and translocation are up-regulated in rice lines accumulating increased NA [11,28,135]. 

## 3. Biotechnological Strategies for Cereal Fe Biofortification

Since some preceding reviews already provide a comprehensive summary of biotechnological Fe biofortification in cereals [5,6,7,8,9], we do not extensively describe different cereal Fe biofortification strategies in this section. Rather, we review below the key achievements by some promising strategies, in association to the mechanism for Fe homeostasis illustrated in the previous section.

### 3.1. Target Fe Increase in Biofortification

Target Fe concentrations for Fe biofortification are 7.5-fold increase from the baseline Fe concentration of around 2 mg/kg in polished rice and twofold increase from the baseline concentration of around 30 mg/kg in whole wheat and maize grains [4].

### 3.2. Fe Biofortification Strategies Involving NAS Overexpression

Overexpression of *NAS* is by far the most common approach in cereal Fe biofortification [91,93,136,137,138,139,140,141]. The reason behind its popularity is the versatile functions of NA in Fe homeostasis in cereal crops (Figure 2). Constitutive *NAS* expression hypothetically affects grain Fe concentration via combination of the following three mechanisms: increased Fe(III)-PS uptake due to enhanced PS synthesis and secretion, increased NA-Fe(II) or PS-Fe(III) translocation from source tissues to grains, and up-regulation of other genes involved in Fe deficiency response (Figure 2). Fe in the grains of *NAS*-overexpressing lines is likely to be chelated by NA (Figure 2), which is a highly bioavailable form [137,142]. Furthermore, *NAS* overexpression allows the inner endosperm to be enriched with Fe [91,93]. This is advantageous for biofortification because outer grain layers are often removed in post-harvest processing. To date, up to 4.5-fold and 2.1-fold increases have been reported in polished rice and whole wheat grains, respectively, in plants overexpressing *NAS* alone [140,141].

### 3.3. Fe Biofortification Strategies Involving Endosperm-Specific FER or VIT Expression

Another classic approach for cereal biofortification is to increase the Fe storage capacity in cereal endosperms. As Fe tends to localize to subcellular organelles for its biochemical function or sequestration in plant cells, increasing the plastid-localized Fe-storage protein FER [143,144,145,146] or tonoplast-localized Fe importer VITs [105] in the endosperm is considered effective for increasing the Fe storage in grains (Figure 2). Fe bound to FER is known to be fairly available for human absorption [147]. Endosperm-specific expression of *FER* alone has led to up to 3.4-fold and around two-fold increase in polished rice and wheat grains, respectively [146,148]. Meanwhile, *VIT* expression in the endosperm resulted in around a twofold increase in wheat and barley whole meal [105].

### 3.4. Combinatory Strategies Lead to Further Increase in Grain Fe Concentration 

In rice, combination of different approaches, especially coupling constitutive *NAS* expression and/or endosperm-specific *FER* expression with other approaches, often leads to higher increase in grain Fe concentration than individual approaches. Enhanced expression of Fe(II)-NA transporter gene *YSL2*, which alone already leads to increased source-to-grain Fe translocation [72], resulted in an up to sixfold increase in combination with *NAS* and *FER* expression (Figure 2) [149]. Moreover, co-expression of *NAS* and *FER* with vacuole Fe exporter gene *NATURAL RESISTANCE-ASSOCIATED MACROPHAGE PROTEIN 3* (*NRAMP3*) from Arabidopsis in aleurone and embryo increased the white rice Fe concentration up to sixfold (Figure 2) [150]. An endosperm Fe concentration increase of 7.5-fold was attained by simply expressing *NAS* and *FER* in an *Indica* rice mega-variety IR64 [151]. By contrast, in wheat, combined expression of *NAS* and *FER* expression did not lead to marked increase in grain Fe concentration, compared to constitutive *NAS* expression alone [140]. 

## 4. Uptake, Transport, and Metabolism of S and Its Biological Functions Related to Metal Homeostasis in Plants

Below, we summarize the mechanisms involved in S homeostasis in plants, deduced mainly from studies on Arabidopsis. Though it is proposed that knowledge obtained through fundamental research on S metabolism in Arabidopsis is directly applicable to crop species [152], it awaits clarification because inter-species variation has been witnessed [153]. 

### 4.1. Sulfate Uptake

Like Fe, S is also a redox-active element [154]. In aerated soil, the bulk of inorganic S is present as sulfate ions (SO_4_^2−^) at a wide range of soil pH [155]. Under anaerobic soil conditions, on the other hand, sulfate is reduced to sulfide forms because microorganisms utilize sulfate as a terminal electron acceptor along with nitrate, manganese (IV), and ferric iron [156,157]. However, in the submerged paddy soil with planted rice, especially in the plant rhizosphere, the concentration of sulfate and/or thiosulfate (which is ultimately oxidized to sulfate) ions is higher than in the flooded soil without rice cultivation [158,159,160]. This relative abundance of oxidized S species in paddy soil with growing plants is ascribed to the oxygen release from the plant roots (radial oxygen loss; ROL) into the rhizosphere [158].

The wide availability of sulfate ions across different soil conditions with vegetation allows the higher plants, including rice and other cereal crops, to take up S in the form of sulfate ions through proton-coupled symporter SULFATE TRANSPORTER (SULTR) family [161,162,163]. The SULTR family in plants is comprised of four distinct subfamilies (SULTR 1–4 subfamilies; Takahashi et al. 2012), among which SULTR1 high-affinity sulfate transporter subfamily plays a central role in sulfate uptake [164,165]. In rice, 3 out of 14 *SULTR* genes are classified as *SULTR1* subfamily genes designated as *OsSULTR 1;1*–*1;3* [166]. In line with its putative function as a sulfate uptake transporter, *OsSULTR1;1* is expressed strongly in the roots [167]. In barley, a high-affinity sulfate transporter SULFATE TRASNPORTER 1 (HvST1), which belongs to the same phylogenic group as SULTR1 in other plant species [167], mediates sulfate uptake from the roots [168,169,170]. Similarly, maize ST1 (ZmST1) has been identified as a sulfate uptake transporter [171,172]. Sulfate transporters expressed in the roots of wheat species under S deficiency are also considered to assume a similar role as SULTR1 transporters in other species, on the basis of the phylogenic similarity to them [173].

### 4.2. Sulfate Long-Distance Transport Via Xylem

Upon its uptake from roots, a large portion of sulfate is delivered to different above-ground organs via xylem [174,175]. Not much is known about the transporters that contribute to the within-root radial sulfate transport and sulfate loading onto the xylem in the roots of cereal species. In Arabidopsis, low-affinity SULTR2;1 and SULTR3;5 transporters are postulated to play a role in optimizing the amount of sulfate loaded onto xylem in root stele [164,176]. In cereals, therefore, SULTR2 and SULTR3 transporters may also have functions analogous to those of *Arabidopsis* homologues. Consistently, *OsSULTR2* genes (*OsSULTR2;1* and *2;2*) and some *OsSULTR3* (*OsSULTR3;5* and *3;6*) genes are expressed at relatively high levels in rice roots [166]. Moreover, both *OsSULTR1;1* and *HvST1* may as well be involved in xylem sulfate loading in roots because they are expressed in root stele of rice and barley, respectively [167,169]. 

### 4.3. S Assimilation and Synthesis of S-Containing Biomolecules Related to Metal Homeostasis

Sulfate is biochemically inert as such [177]. Hence, after its delivery to various tissues in plants, but before its reduction and assimilation, it has to be chemically activated by ATP SULFURYLASE (ATPS) to form adenosine phosphosulfate (APS) [178] (Figure 1). In plants, APS is then reduced by APS REDUCTASE (APSR) to sulfite (SO_3_^2−^), which is further reduced to sulfide (S^2−^) via reaction mediated by SULFITE REDUCTASE (SIR) [179,180] (Figure 1). These sulfate reduction steps are supposed to take place in plastids because the transcripts for the enzymes mediating these reactions harbor sequences coding for plastid transit peptide [178,179,181].

Subsequent to sulfate reduction, sulfide is assimilated into cysteine (Cys) by *O*-ACETYLSERINE (THIOL) LYASE (OAS-TL) in cytosol, plastid, and mitochondria [162,182,183] (Figure 1). In this reaction, sulfide is incorporated into OAS, which is formed through the activation of serine by SERINE ACETYLTRANSFERASE (SAT) [184] (Figure 1). SAT and OAS-TL are known to form a complex referred to as cysteine synthase complex [185]. Cys assumes indispensable biological roles. For example, Cys serves as a ligand for Fe in FeS clusters, which play crucial roles in a range of redox reactions [186,187]. Furthermore, internal Cys level negatively regulates the expression of sulfate uptake transporter genes in cereals [168,170,171].

In addition to its own biological functions, Cys is a common precursor for a variety of S-containing biomolecules in plants, including those involved in metal homeostasis [188]. Methionine (Met) is an important molecule synthesized from Cys via a series of reactions catalyzed by CYSTATHIONINE γ-SYNTHASE (CGS), CYSTATHIONINE β-LYASE (CBL), and Met SYNTHASE (MS) [189] (Figure 1). All three reactions occur in chloroplasts, whereas the final step mediated by MS may occur in the cytoplasm as well [189,190]. Met not only has a basic structural component in plant cells as an amino acid, but also is a biochemical precursor of SAM. SAM plays diverse roles encompassing ethylene biosynthesis, biological methylation, as well as NA synthesis [191] (Figure 1). Though NA itself does not contain S, it is essential for plant metal homeostasis, including Fe homeostasis [192]. 

Furthermore, Cys is required for the synthesis of glutathione (GSH; γ-GluCysGly), which takes part in reactive oxygen species (ROS) scavenging [193,194]. In cereals, GSH can positively affect Cd tolerance via alleviation of ROS damage [195,196,197] (Figure 1). GSH is synthesized from Cys along with glutamate (Glu) and glycine (Gly) by γ-GLUTAMYLCYSTEINE SYNTHETASE (γ-ECS) and GLUTATHIONE SYNTHETASE (GS) [198,199] (Figure 1). The first step of GSH synthesis mediated by γ-ECS is likely to be in plastids, whereas the second reaction owing to GS probably takes place in the cytoplasm [194]. Like Cys, GSH can also negatively regulate S uptake and assimilation processes [168,170,194]. 

At the same time, GSH is also a substrate for the synthesis of phytochelatin (PC), Cys-rich peptide chelators for heavy metals [200], which is mediated by PC SYNTHASE (PCS) [201,202] (Figure 1). In cereals, the role of PCs in Cd detoxification has been reported [202,203,204]. PCs bind to Cd in the cytosol, and the resultant Cd–PC complex is sequestered into vacuoles via ABCC ATP-BINDING CASSETTE TRANSPORTERs (ABCCs) [203]. PCs may also contribute to Zn tolerance in cereals, as Zn can be sequestered into vacuole in a similar manner as Cd [203].

Similar to PCs, METALLOTHIONEINs (MTs) are also Cys-rich polypeptides involved in heavy metal detoxification and homeostasis [200,205]. However, MTs differ from PCs in that they are directly encoded by genes, as opposed to enzymatically synthesized PCs [200] (Figure 1). MTs are found in cytosol and are associated with Zn and Cd tolerance in cereals owing to their capacity to chelate the metals through their thiols [206,207,208,209] (Figure 1). In rice, co-localization of S and Cd in stem nodes have been revealed [77]. At the same time, expression of one of the rice *MT* genes was found to be particularly high in stem nodes [210], coinciding with the notion that MT plays a role in Cd sequestration by binding to it. MTs are also implicated as being involved in the scavenging of ROS caused by excessive Zn and Cd exposure [207,211,212].

Cys enzymatic degradation, for example by L-CYSTEINE DESULFHYDRASE, can lead to the production of hydrogen sulfide (H_2_S) [213]. Though H_2_S has long been recognized as a simply toxic compound, its involvement in stress adaptation, including that to heavy metal stress, has been recently proposed [214].

### 4.4. S Intracellular Homeostasis and Long-Distance Transport Via Phloem

Given that chloroplasts are the main venue for S assimilation in plant cells, transporters to load sulfate to chloroplast are essential for plant S homeostasis. In Arabidopsis, SULTR3 transporters (SULTR3;1–3;4) have been found to be responsible for sulfate uptake by the chloroplast [215]. Furthermore, vacuoles serve as a reservoir for sulfate in plants. In Arabidopsis root cells, SULTR4;1 and SULTR4;2 are identified as sulfate exporters from vacuoles, thereby controlling the amount of sulfate that is stored in roots [216]. Given the general conservation of *SULTR* genes among higher plants [163], cereal homologues of these genes may play similar roles in intracellular sulfate transport. In contrast to sulfate transporters, the identity of transporters mediating intracellular transport of Cys or GSH is not well known [217].

In plants, it is considered that S is transported via phloem primarily in organic forms [218]. *S*-methylmethionine (SMM), derived from Met, has found to be a major form of S in the phloem sap of various plants, including cereals [219]. Besides SMM, GSH is also a form of organic sulfur transported via phloem in cereals [220]. How SMM is loaded onto phloem is yet to be known [217]. As for phloem GSH loading, involvement of OLIGOPEPTIDE TRANSPORTER (OPT) can be expected because OPT6 in Arabidopsis has been identified as a GSH transporter expressed in vasculature [221]. In addition to organic S, sulfate has been also detected in rice phloem sap [222]. As SULTR1;3 in Arabidopsis is characterized as a sulfate transporter likely to be responsible for sulfate loading onto phloem [223], its homologue in cereals may play a similar role.

## 5. Aspects of Fe and S Interaction with Particular Implications for Cereal Fe Biofortification

### 5.1. Effect of Crop S Status on Fe Acquisition and Translocation

Because NA and PS, both of which play a central role in Fe homeostasis in cereal plants, are synthesized from Met, a S-containing amino acid, Fe uptake and translocation in cereal plants are inevitably linked to S metabolism in plants. 

It has been shown that Fe concentration in roots, shoots, or grains of graminaceous species tend to be lower under low external S availability compared to sufficient S availability [224,225,226,227,228,229,230]. The low Fe concentration under little external S availability co-occurs with decreased Fe uptake and/or PS release rates from the roots [225,226]. Moreover, PS synthesis is down-regulated under S deficiency [231]. On the other hand, under Fe-deficient conditions, genes involved in sulfate transport, sulfur assimilation, and methionine metabolism are up-regulated together with the genes in the PS synthesis pathway [26,108,111]. Taken together, sufficient S supply is necessary for the synthesis of PS in cereal crops.

S status of the plant also seems to affect the NA synthesis and Fe distribution in cereal plants. In rice, low external S availability led to a higher amount of NA in roots but lower amount of NA in leaves and stems, in comparison to higher external S conditions [224]. At the same time, Fe concentration in roots and leaves was lower, whereas that in stems was higher in plants exposed to low S environments [224]. In graminaceous species, Fe transferred from roots to shoots first accumulates in stems, and then is delivered to different parts of the shoots, presumably via phloem [74]. Moreover, NA is likely to be at least one of the ligands that chelates Fe in cereal phloem [61,64,71,72]. Therefore, the accumulation of Fe in stems as well as the decrease in leaf Fe concentration can be a result of reduced export of Fe-NA from stems to leaves via phloem, due to less NA availability in shoots in plants under S limitation [224]. Furthermore, increased amount of NA specifically in roots of low-S plants could be a part of the Fe-deficiency response triggered by the reduction in Fe concentration in the leaves. As rice endogenous *NAS* genes are induced particularly strongly in roots but less in shoots under Fe deficiency, the internally available S might have been preferentially allocated to roots for NA synthesis [108,232]. Recently, it was also reported that S limitation leads to decreased level of NA and Fe in rice leaves [229]. All in all, it is likely that insufficient S availability leads to limitation in NA synthesis, thereby disrupting the optimal internal Fe allocation in graminaceous species.

As reviewed above, enhancement of *NAS* expression has been the most popular approach in cereal biofortification studies. Augmented expression of *NAS* in cereals often leads to increased NA and/or PS accumulation in plants or PS secretion from plants [91,93,137,139,141,233,234,235,236]. Given the requirement of S for NA and PS synthesis, it is inferable that cereal lines with increased *NAS* expression demand a larger amount of S than non-transgenic (NT) lines.

Since the 1980s, occurrence of crop S deficiency has increased, resulting from the decreased input of S from the atmosphere and fertilizers into agricultural soils [237,238,239]. Therefore, it would be of realistic interest to assess the grain Fe concentration and S homeostasis in *NAS*-overexpressing lines under varying S availability. If it turns out that S limitation could negatively affect the performance of *NAS*-overexpressing lines, combining *NAS*-overexpression with biotechnological strategies to enhance S uptake, for instance, via overexpression of *SULTR1* transporter genes as has been already demonstrated in Arabidopsis [240], would be a measure that could be attempted.

### 5.2. H_2_S as a Potential Regulator of Crop Fe Homeostasis

Recent evidence shows that H_2_S supply can up-regulate a broad range of Fe acquisition and translocation mechanisms in graminaceous species [241,242] (Figure 1). Although H_2_S has started to be understood as a possible general signaling molecule rather than merely as a phytotoxin [214,243], how it regulates the Fe homeostasis-related machinery is unknown. It is hypothesized that H_2_S affects Fe nutrition though its role as a signaling factor on the biosynthesis of S-containing molecules [241,244].

Modulation of factors that affect a wide range of genes involved in Fe acquisition and translocation has been proven to be useful for increasing grain Fe concentration. For instance, knockdown of *HRZ2* gene, coding for a negative regulatory factor for Fe deficiency response in rice, led to a 2.9-fold increase in polished rice Fe concentration (Figure 2) [122]. Moreover, more than double the concentration of Fe accumulated in unpolished grains in rice overexpressing *IRO2*, which encodes a positive transcription factor for Fe uptake and translocation in rice (Figure 2) [245]. Overexpression of *PRI2* or knockdown of *PRI3* can also lead to increase in brown rice Fe concentration (Figure 2) [125]. Therefore, further investigation into the machinery behind the positive regulation of Fe nutrition by H_2_S may lead to the identification of a regulation factor whose modulation is advantageous for cereal Fe biofortification.

### 5.3. Fe as a Buffer for Sulfide Damage in the Rice Rhizosphere

**Figure 3:**
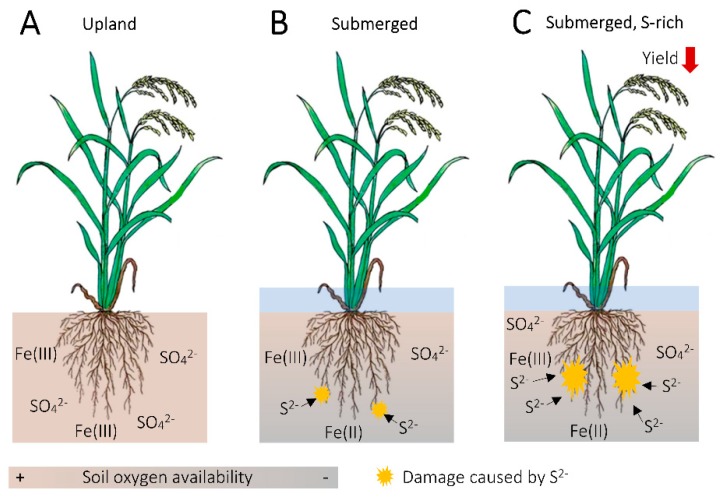
Schematic representation of the occurrence of rice sulfide toxicity as affected by water management practice and soil sulfur (S) availability. (**A**) In upland conditions, the majority of S in soil exists in oxidized forms (SO_4_^2−^) due to high availability of oxygen in the soil. (**B**) Prolonged submergence leads to S reduction in deep soil, where anerobic condition prevails. (**C**) S abundance in the soil can further promote sulfide (S^2−^) occurrence, leading to higher risk of sulfide toxicity in rice, ultimately resulting in yield loss.

Typically, S is reduced to sulfide species including H_2_S in anaerobic rice paddy field soils [155,156] (Figure 3). H_2_S in the rhizosphere can pose toxicity to rice plants, manifested as “Akiochi” (autumn decline) or “Straighthead” disorders [174,246,247]. H_2_S exhibits toxicity by inhibiting cellular respiration, which results in disruption in root growth and nutrient uptake, ultimately leading to yield loss [174,248,249] (Figure 3).

**Figure 4:**
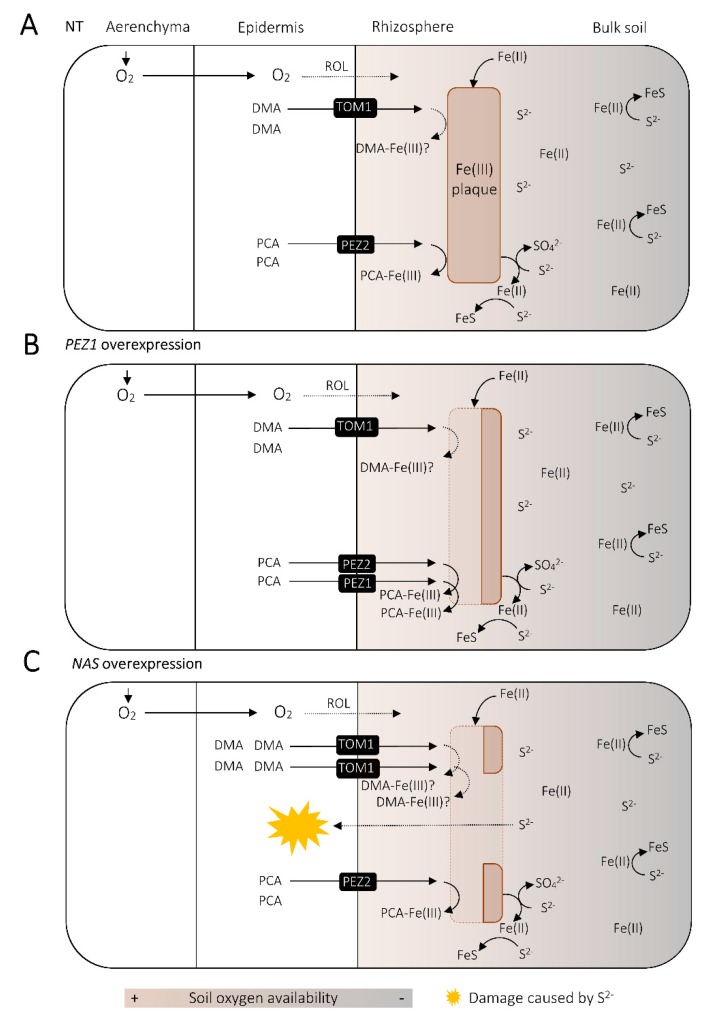
Proposed hypothetical effect of *NICOTIANAMINE SYNTHASE* (*NAS*) overexpression on Fe plaque accumulation and susceptibility to sulfide toxicity. (**A**) In non-transgenic (NT) rice plants, a portion of the oxygen (O_2_) supplied for the roots via aerenchyma diffuses to oxidize the rhizosphere (radial oxygen loss; ROL), where iron (Fe) accumulates as ferric oxides as Fe plaque. Protocatechuic acid (PCA) secreted from PHENOLICS EFFLUX ZERO2 (PEZ2) and possibly deoxymugineic acid (DMA) secreted from TRANSPORTER OF MUGENEIC ACID 1 (TOM1) contribute to solubilization of Fe plaque. Sulfide (S^2−^) can also react with Fe to form FeS. (**B**) *PEZ1*-overexpressing plants reported by Ishimaru et al. (2011) accumulated less Fe on their roots. This was likely due to increased secretion of phenolics to the rhizosphere through PEZ1. (**C**) *NAS* overexpression leads to increased DMA synthesis as well as upregulation of *TOM1*, leading to enhanced secretion of DMA into the rhizosphere. If DMA contributes to Fe plaque solubilization and forms stronger complex with Fe(III) than phenolics do, *NAS* overexpression might result in significant reduction of Fe plaque. This might have implications for buffering sulfide toxicity; however, this needs further research.

Tolerance to H_2_S toxicity is associated with the plant’s capacity to release oxygen into the rhizosphere [250]. Rhizosphere oxygenation due to ROL can oxidize sulfide to sulfate [174]. Moreover, ROL leads to the formation of ferric (hydr)oxide deposition, called Fe plaque, on the root surface of hydrophyte species, which can buffer H_2_S toxicity by reacting with H_2_S to form insoluble FeS (Figure 4) [174,247,251,252]. In agreement, H_2_S damage often becomes evident towards the grain maturity [246,249], during which the rice rhizosphere oxidization capacity due to ROL goes down and Fe plaque on the roots becomes less prominent [253]. Moreover, Fe plaque on the rice roots decreases under excessive S application to the soil, which probably resulted in increased sulfide level in the rhizosphere [230,254], further advocating the role of Fe plaque to soften the potential sulfide damage to the roots.

It is unclear whether and to what degree increased PS release from biotechnologically Fe-biofortified rice lines affect the formation and accumulation of Fe plaque on their roots. Some studies have compared the amount or integrity of root Fe plaque on NT plants with contrasting Fe status and PS release; however, the insights they offer are rather dividing. Zhang et al. (1999) reported that the amount of root Fe plaque did not change between rice plants with different Fe status and PS secretion rates [255]. In contrast, other studies have shown that the rice plants with high PS release can take up larger amount of elements adsorbed on Fe plaque than those with low PS secretion, which implies that increased PS release at least negatively affects the integrity of root Fe plaque [256,257]. Nonetheless, these observations may not necessarily help us understand the effect of PS on Fe plaque accumulation on Fe-biofortified rice lines to be grown in actual rice field soils, because these studies employed not soil but nutrient solutions as the substrate for plant growth, which must have allowed constant supply of Fe from the surrounding solution into the rhizosphere solution to form Fe plaque. Moreover, it has been demonstrated that NT rice plants regulate PS secretion and the expression of genes underlying PS synthesis in a diurnal manner, even under Fe deficiency [28,258]. On the contrary, some Fe-biofortified rice lines, such as those with constitutive *NAS* expression, synthesize and release increased PS [28,138], putatively in a constant manner without circadian oscillation. Therefore, it remains an open question as to whether Fe-biofortified rice lines would follow the same pattern of Fe plaque accumulation as the NT plants with low Fe status.

Though also being a study utilizing a nutrient solution for plant husbandry, a report by Ishimaru et al. (2011) provides a rough idea as to what extent constitutively increased secretion of an Fe-solubilizing agent from rice roots can impact the formation of Fe plaque. In this research, they overexpressed *PEZ1*, which encodes a transporter that exports PCA into the apoplastic region to solubilize the precipitated Fe [84]. As a result, Fe deposition on the root surface of *PEZ1*-overexpressing line decreased notably compared to that of NT plants, when grown in an Fe-rich nutrient solution [84] (Figure 4). The stability constant of Fe(III)-PCA is reportedly lower than that of Fe(III)-DMA [259,260]. Thus, it is not surprising if abundant PS secretion by *NAS*-overexpression lines results in significantly reduced amount of Fe plaque in some growth conditions, potentially rendering them more prone to H_2_S toxicity in the roots than NT plants (Figure 4). In conclusion, studies have to be made in the future to assess the susceptibility to H_2_S damage of the Fe-biofortified rice lines, performed in an experimental system with soils with different S input as well as Fe content, given that one of the major factors that contribute to rice H_2_S toxicity is the presence of a high amount of S in the soil [247] (Figure 3) and that soil Fe content can also affect the occurrence of H_2_S toxicity, as seen in acid sulfate soils often found in rice cultivation systems in the tropics [261,262]. 

### 5.4. Roles of S-Containing Biomolecules in Response to Concomitant Increase in Heavy Metal Uptake by Fe-Biofortified Crops

Very frequently, biotechnological Fe biofortification of cereal crops results in concomitant accumulation of Zn in grains and other organs [91,137,139,150,263], due to partially shared molecular mechanisms underlying the homeostasis of these two metal elements [12] (Figure 1). Like Fe, Zn is toxic when present in excess [264,265]. In barley seed transfer cells, increased Zn loading alone or combined with Fe loading to the grain triggers a major alternation in transcriptome [266]. It is postulated that the metabolic cost associated with such a transcriptomic re-organization may limit the metal translocation to the grains or compromise yield [266]. In agreement, some *NAS*-overexpressing rice lines exhibit grain yield loss, which is associated with high grain Zn concentration, suggesting the link between the yield decrease and the Zn homeostasis disruption in Fe-biofortified plants [10].

Rice is sometimes grown in fields contaminated with Cd [267]. Moreover, rice can take up Cd via IRT transporters [268], and a rice line overexpressing *IRT1* gene indeed exhibits increased sensitivity to Cd, as well as to Zn [269]. Considering the fact that endogenous *IRT1* expression is up-regulated in shoots and/or roots in rice lines overexpressing *NAS* or overaccumulating NA (Figure 2) [11,135,150], there remains a possibility that *NAS*-overexpressing rice lines are more susceptible to Cd and/or Zn stress. However, this aspect remains underexplored in the iron-biofortified crops developed so far.

In the seed transfer cells of barley plants with foliar Zn application, a wide range of stress-related genes, including *MT* genes and genes involved in GSH synthesis (and probably the downstream PC synthesis), are notably up-regulated [266,270]. This implies a major role of thiol-containing molecules in response to potentially increased heavy metal stress in Fe-biofortified crops (Figure 1). Clarification of the extent of heavy metal stress in Fe-biofortified crops, in tandem with the further understanding of the exact role of S-containing biomolecules in coping with such a stress, may provide an insight that is useful for future cereal Fe biofortification efforts.

Together, there are various aspects of interaction between Fe and S in cereals that are relevant and potentially important for cereal Fe biofortification studies. We propose that future characterization of Fe-biofortified cereal lines, especially that in the field, take into account the aspects of Fe and S interaction reviewed above in order to advance our understanding of Fe-biofortified crops. 

## References

[1] McLean E., Cogswell M., Egli I., Wojdyla D., De Benoist B. (2009). Worldwide prevalence of anaemia, WHO Vitamin and Mineral Nutrition Information System, 1993–2005. Public Health Nutr..

[2] WHO (2015). The Global Prevalence of Anemia in 2011.

[3] Graham R.D., Welch R.M., Bouis H.E. (2001). Addressing micronutrient malnutrition through enhancing the nutritional quality of staple foods: Principles, perspectives and knowledge gaps. Adv. Agron..

[4] Bouis H.E., Welch R.M. (2010). Biofortification—A sustainable agricultural strategy for reducing micronutrient malnutrition in the Global South. Crop Sci..

[5] Connorton J.M., Balk J. (2019). Iron biofortification of staple crops: Lessons and challenges in plant genetics. Plant Cell Physiol..

[6] Masuda H., Aung M.S., Nishizawa N.K. (2013). Iron biofortification of rice using different transgenic approaches. Rice.

[7] Slamet-Loedin I.H., Johnson-Beebout S.E., Impa S., Tsakirpaloglou N. (2015). Enriching rice with Zn and Fe while minimizing Cd risk. Front. Plant Sci..

[8] Ludwig Y., Slamet-Loedin I.H. (2019). Genetic biofortification to enrich rice and wheat grain iron: From genes to product. Front. Plant Sci..

[9] Kawakami Y., Bhullar N.K. (2018). Molecular processes in iron and zinc homeostasis and their modulation for biofortification in rice. J. Integr. Plant Biol..

[10] Moreno-Moyano L.T., Bonneau J.P., Sánchez-Palacios J.T., Tohme J., Johnson A.A.T. (2016). Association of increased grain iron and zinc concentrations with agro-morphological traits of biofortified rice. Front. Plant Sci..

[11] Wang M., Gruissem W., Bhullar N.K. (2013). Nicotianamine synthase overexpression positively modulates iron homeostasis-related genes in high iron rice. Front. Plant Sci..

[12] Kobayashi T., Nishizawa N.K. (2012). Iron uptake, translocation, and regulation in higher plants. Annu. Rev. Plant Biol..

[13] Bashir K., Nozoye T., Ishimaru Y., Nakanishi H., Nishizawa N.K. (2013). Exploiting new tools for iron bio-fortification of rice. Biotechnol. Adv..

[14] Connorton J.M., Balk J., Rodríguez-Celma J. (2017). Iron homeostasis in plants—A brief overview. Metallomics.

[15] Römheld V., Marschner H. (1986). Evidence for a specific uptake system for iron phytosiderophores in roots of grasses. Plant Physiol..

[16] Takagi S. (1976). Naturally occurring iron-chelating compounds in oat- and rice-root washings. Soil Sci. Plant Nutr..

[17] Nozoye T., Nagasaka S., Kobayashi T., Takahashi M., Sato Y., Sato Y., Uozumi N., Nakanishi H., Nishizawa N.K. (2011). Phytosiderophore efflux transporters are crucial for iron acquisition in graminaceous plants. J. Biol. Chem..

[18] Murata Y., Ma J.F., Yamaji N., Ueno D., Nomoto K., Iwashita T. (2006). A specific transporter for iron(III)-phytosiderophore in barley roots. Plant J..

[19] Curie C., Panaviene Z., Loulergue C., Dellaporta S.L., Briat J.F., Walker E.L. (2001). Maize *yellow stripe1* encodes a membrane protein directly involved in Fe(III) uptake. Nature.

[20] Inoue H., Kobayashi T., Nozoye T., Takahashi M., Kakei Y., Suzuki K., Nakazono M., Nakanishi H., Mori S., Nishizawa N.K. (2009). Rice OsYSL15 is an iron-regulated iron (III)-deoxymugineic acid transporter expressed in the roots and is essential for iron uptake in early growth of the seedlings. J. Biol. Chem..

[21] Lee S., Chiecko J.C., Kim S.A., Walker E.L., Lee Y., Guerinot M.L., An G. (2009). Disruption of *OsYSL15* leads to iron inefficiency in rice plants. Plant Physiol..

[22] Mori S., Nishizawa N. (1987). Methionine as a dominant precursor of phytosiderophores in *Graminaceae* plants. Plant Cell Physiol..

[23] Shojima S., Nishizawa N.-K., Fushiya S., Nozoe S., Irifune T., Mori S. (1990). Biosynthesis of phytosiderophores. Plant Physiol..

[24] Ma J.F., Shinada T., Matsuda C., Nomoto K. (1995). Biosynthesis of phytosiderophores, mugineic acids, associated with methionine cycling. J. Biol. Chem..

[25] Nishizawa N., Mori S. (1987). The particular vesicle appearing in barley root cells and its relation to mugineic acid secretion. J. Plant Nutr..

[26] Negishi T., Nakanishi H., Yazaki J., Kishimoto N., Fujii F., Shimbo K., Yamamoto K., Sakata K., Sasaki T., Kikuchi S. (2002). cDNA microarray analysis of gene expression during Fe-deficiency stress in barley suggests that polar transport of vesicles is implicated in phytosiderophore secretion in Fe-deficient barley roots. Plant J..

[27] Mizuno D., Higuchi K., Sakamoto T., Nakanishi H., Mori S., Nishizawa N.K. (2003). Three nicotianamine synthase genes isolated from maize are differentially regulated by iron nutritional status. Plant Physiol..

[28] Nozoye T., Nagasaka S., Bashir K., Takahashi M., Kobayashi T., Nakanishi H., Nishizawa N.K. (2014). Nicotianamine synthase 2 localizes to the vesicles of iron-deficient rice roots, and its mutation in the YXXφ or LL motif causes the disruption of vesicle formation or movement in rice. Plant J..

[29] Nozoye T., Tsunoda K., Nagasaka S., Bashir K., Takahashi M., Kobayashi T., Nakanishi H., Nishizawa N.K. (2014). Rice nicotianamine synthase localizes to particular vesicles for proper function. Plant Signal. Behav..

[30] Zheng L., Fujii M., Yamaji N., Sasaki A., Yamane M., Sakurai I., Sato K., Ma J.F. (2011). Isolation and characterization of a barley yellow stripe-like gene, HvYSL5. Plant Cell Physiol..

[31] Higuchi K., Suzuki K., Nakanishi H., Yamaguchi H., Nishizawa N.-K., Mori S. (1999). Cloning of nicotianamine synthase genes, novel genes involved in the biosynthesis of phytosiderophores. Plant Physiol..

[32] Takahashi M., Yamaguchi H., Nakanishi H., Shioiri T., Nishizawa N.K., Mori S. (1999). Cloning two genes for nicotianamine aminotransferase, a critical enzyme in iron acquisition (Strategy II) in graminaceous plants. Plant Physiol..

[33] Bashir K., Inoue H., Nagasaka S., Takahashi M., Nakanishi H., Mori S., Nishizawa N.K. (2006). Cloning and characterization of deoxymugineic acid synthase genes from graminaceous plants. J. Biol. Chem..

[34] Kawai S., Takagi S.I., Sato Y. (1988). Mugineic acid-family phytosiderophores in root-secretions of barley, corn and sorghum varieties. J. Plant Nutr..

[35] Ma J.F., Taketa S., Chang Y.-C., Takeda K., Matsumoto H. (1999). Biosynthesis of phytosiderophores in several Triticeae species with different genomes. J. Exp. Bot..

[36] Nakanishi H., Yamaguchi H., Sasakuma T., Nishizawa N.K., Mori S. (2000). Two dioxygenase genes, *Ids3* and *Ids2*, from *Hordeum vulgare* are involved in the biosynthesis of mugineic acid family phytosiderophores. Plant Mol. Biol..

[37] von Wirén N., Khodr H., Hider R.C. (2002). Hydroxylated phytosiderophore species possess an enhanced chelate stability and affinity for Iron(III). Plant Physiol..

[38] Ueno D., Rombolà A.D., Iwashita T., Nomoto K., Ma J.F. (2007). Identification of two novel phytosiderophores secreted by perennial grasses. New Phytol..

[39] Nozoye T., Aung M.S., Masuda H., Nakanishi H., Nishizawa N.K. (2017). Bioenergy grass [*Erianthus ravennae* (L.) Beauv.] secretes two members of mugineic acid family phytosiderophores which involved in their tolerance to Fe deficiency. Soil Sci. Plant Nutr..

[40] Takagi S.-I., Barton L., Hemming B.C. (1993). Production of phytosiderophores. Iron Chelation in Plants and Soil Microorganisms.

[41] Ishimaru Y., Suzuki M., Tsukamoto T., Suzuki K., Nakazono M., Kobayashi T., Wada Y., Watanabe S., Matsuhashi S., Takahashi M. (2006). Rice plants take up iron as an Fe^3+^-phytosiderophore and as Fe^2+^. Plant J..

[42] Wairich A., de Oliveira B.H.N., Arend E.B., Duarte G.L., Ponte L.R., Sperotto R.A., Ricachenevsky F.K., Fett J.P. (2019). The Combined Strategy for iron uptake is not exclusive to domesticated rice (*Oryza sativa*). Sci. Rep..

[43] Wang P., Yamaji N., Inoue K., Mochida K., Ma J.F. (2020). Plastic transport systems of rice for mineral elements in response to diverse soil environmental changes. New Phytol..

[44] Zaharieva T., Römheld V. (2000). Specific Fe^2+^ uptake system in strategy I plants inducible under Fe deficiency. J. Plant Nutr..

[45] Bughio N., Yamaguchi H., Nishizawa N.K., Nakanishi H., Mori S. (2002). Cloning an iron-regulated metal transporter from rice. J. Exp. Bot..

[46] Liu C., Gao T., Liu Y., Liu J., Li F., Chen Z., Li Y., Lv Y., Song Z., Reinfelder J.R. (2019). Isotopic fingerprints indicate distinct strategies of Fe uptake in rice. Chem. Geol..

[47] Enstone D.E., Peterson C.A., Ma F. (2002). Root endodermis and exodermis: Structure, function, and responses to the environment. J. Plant Growth Regul..

[48] Perumalla C.J., Peterson C.A., Enstone D.E. (1990). A survey of angiosperm species to detect hypodermal Casparian bands. I. Roots with a uniseriate hypodermis and epidermis. Bot. J. Linn. Soc..

[49] Peterson C.A., Emanuel M.E., Wilson C. (1982). Identification of a Casparian band in the hypodermis of onion and corn roots. Can. J. Bot..

[50] Clark L.H., Harris W.H. (1981). Observations on the root anatomy of rice (*Oryza sativa* L.). Am. J. Bot..

[51] Tylová E., Pecková E., Blascheová Z., Soukup A. (2017). Casparian bands and suberin lamellae in exodermis of lateral roots: An important trait of roots system response to abiotic stress factors. Ann. Bot..

[52] Kreszies T., Eggels S., Kreszies V., Osthoff A., Shellakkutti N., Baldauf J.A., Zeisler-Diehl V.V., Hochholdinger F., Ranathunge K., Schreiber L. (2020). Seminal roots of wild and cultivated barley differentially respond to osmotic stress in gene expression, suberization, and hydraulic conductivity. Plant Cell Environ..

[53] Ueno D., Yamaji N., Ma J.F. (2009). Further characterization of ferric—Phytosiderophore transporters ZmYS1 and HvYS1 in maize and barley. J. Exp. Bot..

[54] Araki R., Murata J., Murata Y. (2011). A novel barley yellow stripe 1-like transporter (HvYSL2) localized to the root endodermis transports metal-phytosiderophore complexes. Plant Cell Physiol..

[55] Jackson M.B., Armstrong W. (1999). Formation of aerenchyma and the processes of plant ventilation in relation to soil flooding and submergence. Plant Biol..

[56] Kawai M., Samarajeewa P.K., Barrero R.A., Nishiguchi M., Uchimiya H. (1998). Cellular dissection of the degradation pattern of cortical cell death during aerenchyma formation of rice roots. Planta.

[57] Sasaki A., Yamaji N., Ma J.F. (2016). Transporters involved in mineral nutrient uptake in rice. J. Exp. Bot..

[58] Che J., Yamaji N., Ma J.F. (2018). Efficient and flexible uptake system for mineral elements in plants. New Phytol..

[59] Ogo Y., Kakei Y., Itai R.N., Kobayashi T., Nakanishi H., Takahashi H., Nakazono M., Nishizawa N.K. (2014). Spatial transcriptomes of iron-deficient and cadmium-stressed rice. New Phytol..

[60] Hell R., Stephan U.W. (2003). Iron uptake, trafficking and homeostasis in plants. Planta.

[61] von Wirén N., Klair S., Bansal S., Briat J.-F., Khodr H., Shioiri T., Leigh R.A., Hider R.C. (1999). Nicotianamine chelates both FeIII and FeII. Implications for metal transport in Plants. Plant Physiol..

[62] Ariga T., Hazama K., Yanagisawa S., Yoneyama T. (2014). Chemical forms of iron in xylem sap from graminaceous and non-graminaceous plants. Soil Sci. Plant Nutr..

[63] Kakei Y., Yamaguchi I., Kobayashi T., Takahashi M., Nakanishi H., Yamakawa T., Nishizawa N.K. (2009). A highly sensitive, quick and simple quantification method for nicotianamine and 2′-deoxymugineic acid from minimum samples using LC/ESI-TOF-MS achieves functional analysis of these components in plants. Plant Cell Physiol..

[64] Rellán-Álvarez R., Abadía J., Álvarez-Fernández A. (2008). Formation of metal-nicotianamine complexes as affected by pH, ligand exchange with citrate and metal exchange. A study by electrospray ionization time-of-flight mass spectrometry. Rapid Commun. Mass Spectrom..

[65] Yokosho K., Yamaji N., Ueno D., Mitani N., Ma J.F. (2009). OsFRDL1 is a citrate transporter required for efficient translocation of iron in rice. Plant Physiol..

[66] Nishiyama R., Kato M., Nagata S., Yanagisawa S., Yoneyama T. (2012). Identification of Zn-nicotianamine and Fe-2’-deoxymugineic acid in the phloem sap from rice plants (*Oryza sativa* L.). Plant Cell Physiol..

[67] Alam S., Kamei S., Kawai S. (2001). Effect of iron deficiency on the chemical composition of the xylem sap of barley. Soil Sci. Plant Nutr..

[68] Mori S., Nishizawa N., Hayashi H., Chino M., Yoshimura E., Ishihara J. (1991). Why are young rice plants highly susceptible to iron deficiency?. Plant Soil.

[69] Nozoye T., Nagasaka S., Kobayashi T., Sato Y., Uozumi N., Nakanishi H., Nishizawa N.K. (2015). The phytosiderophore efflux transporter TOM2 is involved in metal transport in Rice. J. Biol. Chem..

[70] Nozoye T., von Wirén N., Sato Y., Higashiyama T., Nakanishi H., Nishizawa N.K. (2019). Characterization of the nicotianamine exporter ENA1 in rice. Front. Plant Sci..

[71] Koike S., Inoue H., Mizuno D., Takahashi M., Nakanishi H., Mori S., Nishizawa N.K. (2004). OsYSL2 is a rice metal-nicotianamine transporter that is regulated by iron and expressed in the phloem. Plant J..

[72] Ishimaru Y., Masuda H., Bashir K., Inoue H., Tsukamoto T., Takahashi M., Nakanishi H., Aoki N., Hirose T., Ohsugi R. (2010). Rice metal-nicotianamine transporter, OsYSL2, is required for the long-distance transport of iron and manganese. Plant J..

[73] Aoyama T., Kobayashi T., Takahashi M., Nagasaka S., Usuda K., Kakei Y., Ishimaru Y., Nakanishi H., Mori S., Nishizawa N.K. (2009). OsYSL18 is a rice iron(III)-deoxymugineic acid transporter specifically expressed in reproductive organs and phloem of lamina joints. Plant Mol. Biol..

[74] Tsukamoto T., Nakanishi H., Uchida H., Watanabe S., Matsuhashi S., Mori S., Nishizawa N.K. (2009). ^52^Fe translocation in barley as monitored by a positron-emitting tracer imaging system (PETIS): Evidence for the direct translocation of Fe from roots to young leaves via phloem. Plant Cell Physiol..

[75] Yamaji N., Ma J.F. (2014). The node, a hub for mineral nutrient distribution in graminaceous plants. Trends Plant Sci..

[76] Yamaji N., Ma J.F. (2017). Node-controlled allocation of mineral elements in Poaceae. Curr. Opin. Plant Biol..

[77] Yamaguchi N., Ishikawa S., Abe T., Baba K., Arao T., Terada Y. (2012). Role of the node in controlling traffic of cadmium, zinc, and manganese in rice. J. Exp. Bot..

[78] Moore K.L., Chen Y., van de Meene A.M.L., Hughes L., Liu W., Geraki T., Mosselmans F., Mcgrath S.P., Grovenor C., Zhao F.J. (2014). Combined NanoSIMS and synchrotron X-ray fluorescence reveal distinct cellular and subcellular distribution patterns of trace elements in rice tissues. New Phytol..

[79] Yamaji N., Ma J.F. (2019). Bioimaging of multiple elements by high-resolution LA-ICP-MS reveals altered distribution of mineral elements in the nodes of rice mutants. Plant J..

[80] Yokosho K., Yamaji N., Ma J.F. (2016). *OsFRDL1* expressed in nodes is required for distribution of iron to grains in rice. J. Exp. Bot..

[81] Bienfait H.F., van den Briel W., Mesland-Mul N.T. (1985). Free space iron pools in roots: Generation and mobilization. Plant Physiol..

[82] Zhang F., Römheld V., Marschner H. (1991). Role of the root apoplasm for iron acquisition by wheat plants. Plant Physiol..

[83] Shi R., Melzer M., Zheng S., Benke A., Stich B., von Wirén N. (2018). Iron retention in root hemicelluloses causes genotypic variability in the tolerance to iron deficiency-induced chlorosis in maize. Front. Plant Sci..

[84] Ishimaru Y., Kakei Y., Shimo H., Bashir K., Sato Y., Sato Y., Uozumi N., Nakanishi H., Nishizawa N.K. (2011). A rice phenolic efflux transporter is essential for solubilizing precipitated apoplasmic iron in the plant stele. J. Biol. Chem..

[85] Bashir K., Ishimaru Y., Shimo H., Kakei Y., Senoura T., Takahashi R., Sato Y., Sato Y., Uozumi N., Nakanishi H. (2011). Rice phenolics efflux transporter 2 (PEZ2) plays an important role in solubilizing apoplasmic iron. Soil Sci. Plant Nutr..

[86] Palmgren M.G., Clemens S., Williams L.E., Krämer U., Borg S., Schjørring J.K., Sanders D. (2008). Zinc biofortification of cereals: Problems and solutions. Trends Plant Sci..

[87] Zee S., O’brien T. (1970). A special type of tracheary element associated with “xylem discontinuity” in the floral axis of wheat. Aust. J. Biol. Sci..

[88] Stomph T.J., Jiang W., Struik P.C. (2009). Zinc biofortification of cereals: Rice differs from wheat and barley. Trends Plant Sci..

[89] Zee S.-Y. (1972). Vascular tissue and transfer cell distribution in the rice spikelet. Aust. J. Biol. Sci..

[90] Wu X., Liu J., Li D., Liu C.M. (2016). Rice caryopsis development I: Dynamic changes in different cell layers. J. Integr. Plant Biol..

[91] Beasley J.T., Bonneau J.P., Sánchez-Palacios J.T., Moreno-Moyano L.T., Callahan D.L., Tako E., Glahn R.P., Lombi E., Johnson A.A.T. (2019). Metabolic engineering of bread wheat improves grain iron concentration and bioavailability. Plant Biotechnol. J..

[92] Kyriacou B., Moore K.L., Paterson D., De Jonge M.D., Howard D.L., Stangoulis J., Tester M., Lombi E., Johnson A.A.T. (2014). Localization of iron in rice grain using synchrotron X-ray fluorescence microscopy and high resolution secondary ion mass spectrometry. J. Cereal Sci..

[93] Johnson A.A.T., Kyriacou B., Callahan D.L., Carruthers L., Stangoulis J., Lombi E., Tester M. (2011). Constitutive overexpression of the *OsNAS* gene family reveals single-gene strategies for effective iron- and zinc-biofortification of rice endosperm. PLoS ONE.

[94] Senoura T., Sakashita E., Kobayashi T., Takahashi M., Aung M.S., Masuda H., Nakanishi H., Nishizawa N.K. (2017). The iron-chelate transporter OsYSL9 plays a role in iron distribution in developing rice grains. Plant Mol. Biol..

[95] Thomine S., Vert G. (2013). Iron transport in plants: Better be safe than sorry. Curr. Opin. Plant Biol..

[96] Nouet C., Motte P., Hanikenne M. (2011). Chloroplastic and mitochondrial metal homeostasis. Trends Plant Sci..

[97] Vigani G., Solti Á., Thomine S., Philippar K. (2019). Essential and detrimental—An update on intracellular iron trafficking and homeostasis. Plant Cell Physiol..

[98] Bashir K., Ishimaru Y., Shimo H., Nagasaka S., Fujimoto M., Takanashi H., Tsutsumi N., An G., Nakanishi H., Nishizawa N.K. (2011). The rice mitochondrial iron transporter is essential for plant growth. Nat. Commun..

[99] Han J.H., Song X.F., Li P., Yang H.J., Yin L.P. (2009). Maize ZmFDR3 localized in chloroplasts is involved in iron transport. Sci. China, Ser. C Life Sci..

[100] Zhang X.-Y., Zhang X., Zhang Q., Pan X.-X., Yan L.-C., Ma X.-J., Zhao W.-Z., Qi X.-T., Yin L.-P. (2017). *Zea mays* Fe deficiency-related 4 (ZmFDR4) functions as an iron transporter in the plastids of monocots. Plant J..

[101] Fobis-Loisy I., Loridon K., Lobreaux S., Lebrun M., Briat J.-F. (1995). Structure and differential expression of two maize ferritin genes in response to iron and abscisic acid. Eur. J. Biochem..

[102] Stein R.J., Ricachenevsky F.K., Fett J.P. (2009). Differential regulation of the two rice ferritin genes (*OsFER1 and OsFER2*). Plant Sci..

[103] Borg S., Brinch-Pedersen H., Tauris B., Madsen L.H., Darbani B., Noeparvar S., Holm P.B. (2012). Wheat ferritins: Improving the iron content of the wheat grain. J. Cereal Sci..

[104] Zhang Y., Xu Y.H., Yi H.Y., Gong J.M. (2012). Vacuolar membrane transporters OsVIT1 and OsVIT2 modulate iron translocation between flag leaves and seeds in rice. Plant J..

[105] Connorton J.M., Jones E.R., Rodríguez-Ramiro I., Fairweather-Tait S., Uauy C., Balk J. (2017). Wheat vacuolar iron transporter TaVIT2 transports Fe and Mn and is effective for biofortification. Plant Physiol..

[106] Che J., Yokosho K., Yamaji N., Ma J.F. (2019). A vacuolar phytosiderophore transporter alters iron and zinc accumulation in polished rice grains. Plant Physiol..

[107] Li L., Ye L., Kong Q., Shou H. (2019). A vacuolar membrane ferric-chelate reductase, OsFRO1, alleviates Fe toxicity in rice (*Oryza sativa* L.). Front. Plant Sci..

[108] Kobayashi T., Suzuki M., Inoue H., Itai R.N., Takahashi M., Nakanishi H., Mori S., Nishizawa N.K. (2005). Expression of iron-acquisition-related genes in iron-deficient rice is co-ordinately induced by partially conserved iron-deficiency-responsive elements. J. Exp. Bot..

[109] Li Y., Wang N., Zhao F., Song X., Yin Z., Huang R., Zhang C. (2014). Changes in the transcriptomic profiles of maize roots in response to iron-deficiency stress. Plant Mol. Biol..

[110] Wang M., Kawakami Y., Bhullar N.K. (2019). Molecular Analysis of Iron Deficiency Response in Hexaploid Wheat. Front. Sustain. Food Syst..

[111] Nagasaka S., Takahashi M., Nakanishi-Itai R., Bashir K., Nakanishi H., Mori S., Nishizawa N.K. (2009). Time course analysis of gene expression over 24 hours in Fe-deficient barley roots. Plant Mol. Biol..

[112] Kobayashi T., Nishizawa N.K. (2014). Iron sensors and signals in response to iron deficiency. Plant Sci..

[113] Kobayashi T., Ogo Y., Itai R.N., Nakanishi H., Takahashi M., Mori S., Nishizawa N.K. (2007). The transcription factor IDEF1 regulates the response to and tolerance of iron deficiency in plants. Proc. Natl. Acad. Sci. USA.

[114] Kobayashi T., Itai R.N., Ogo Y., Kakei Y., Nakanishi H., Takahashi M., Nishizawa N.K. (2009). The rice transcription factor IDEF1 is essential for the early response to iron deficiency, and induces vegetative expression of late embryogenesis abundant genes. Plant J..

[115] Kobayashi T., Itai R.N., Aung M.S., Senoura T., Nakanishi H., Nishizawa N.K. (2012). The rice transcription factor IDEF1 directly binds to iron and other divalent metals for sensing cellular iron status. Plant J..

[116] Kobayashi T., Nakayama Y., Itai R.N., Nakanishi H., Yoshihara T., Mori S., Nishizawa N.K. (2003). Identification of novel cis-acting elements, IDE1 and IDE2, of the barley *IDS2* gene promoter conferring iron-deficiency-inducible, root-specific expression in heterogeneous tobacco plants. Plant J..

[117] Zhang L., Nakanishi Itai R., Yamakawa T., Nakanishi H., Nishizawa N.K., Kobayashi T. (2014). The Bowman-Birk trypsin inhibitor IBP1 interacts with and prevents degradation of IDEF1 in rice. Plant Mol. Biol. Report..

[118] Kobayashi T., Ogo Y., Aung M.S., Nozoye T., Itai R.N., Nakanishi H., Yamakawa T., Nishizawa N.K. (2010). The spatial expression and regulation of transcription factors IDEF1 and IDEF2. Ann. Bot..

[119] Ogo Y., Itai R.N., Nakanishi H., Inoue H., Kobayashi T., Suzuki M., Takahashi M., Mori S., Nishizawa N.K. (2006). Isolation and characterization of IRO2, a novel iron-regulated bHLH transcription factor in graminaceous plants. J. Exp. Bot..

[120] Ogo Y., Nakanishi Itai R., Nakanishi H., Kobayashi T., Takahashi M., Mori S., Nishizawa N.K. (2007). The rice bHLH protein OsIRO2 is an essential regulator of the genes involved in Fe uptake under Fe-deficient conditions. Plant J..

[121] Wang S., Li L., Ying Y., Wang J., Shao J.F., Yamaji N., Whelan J., Ma J.F., Shou H. (2019). A transcription factor OsbHLH156 regulates Strategy II iron acquisition through localising IRO2 to the nucleus in rice. New Phytol..

[122] Kobayashi T., Nagasaka S., Senoura T., Itai R.N., Nakanishi H., Nishizawa N.K. (2013). Iron-binding haemerythrin RING ubiquitin ligases regulate plant iron responses and accumulation. Nat. Commun..

[123] Zhang H., Li Y., Yao X., Liang G., Yu D. (2017). POSITIVE REGULATOR OF IRON HOMEOSTASIS1, OSPRI1, facilitates Iron homeostasis. Plant Physiol..

[124] Zhang H., Li Y., Pu M., Xu P., Liang G., Yu D. (2020). *Oryza sativa* POSITIVE REGULATOR OF IRON DEFICIENCY RESPONSE 2 (OsPRI2) and OsPRI3 are involved in the maintenance of Fe homeostasis. Plant. Cell Environ..

[125] Kobayashi T., Ozu A., Kobayashi S., An G., Seong J. (2019). OsbHLH058 and OsbHLH059 transcription factors positively regulate iron deficiency responses in rice. Plant Mol. Biol..

[126] Zheng L., Ying Y., Wang L., Wang F., Whelan J., Shou H. (2010). Identification of a novel iron regulated basic helix-loop-helix protein involved in Fe homeostasis in *Oryza sativa*. BMC Plant Biol..

[127] Ogo Y., Kobayashi T., Nakanishi Itai R., Nakanishi H., Kakei Y., Takahashi M., Toki S., Mori S., Nishizawa N.K. (2008). A novel NAC transcription factor, IDEF2, that recognizes the Iron Deficiency-responsive Element 2 regulates the genes involved in iron homeostasis in plants. J. Biol. Chem..

[128] Wang L., Ying Y., Narsai R., Ye L., Zheng L., Tian J., Whelan J., Shou H. (2013). Identification of OsbHLH133 as a regulator of iron distribution between roots and shoots in *Oryza sativa*. Plant. Cell Environ..

[129] Grillet L., Lan P., Li W., Mokkapati G., Schmidt W. (2018). IRON MAN is a ubiquitous family of peptides that control iron transport in plants. Nat. Plants.

[130] Uauy C., Distelfeld A., Fahima T., Blechl A., Dubcovsky J. (2006). A NAC Gene regulating senescence improves grain protein, zinc, and iron content in wheat. Science.

[131] Distelfeld A., Pearce S.P., Avni R., Scherer B., Uauy C., Piston F., Slade A., Zhao R., Dubcovsky J. (2012). Divergent functions of orthologous NAC transcription factors in wheat and rice. Plant Mol. Biol..

[132] Kobayashi T., Itai R.N., Senoura T., Oikawa T., Ishimaru Y., Ueda M., Nakanishi H., Nishizawa N.K. (2016). Jasmonate signaling is activated in the very early stages of iron deficiency responses in rice roots. Plant Mol. Biol..

[133] Shen C., Yue R., Sun T., Zhang L., Yang Y., Wang H. (2015). OsARF16, a transcription factor regulating auxin redistribution, is required for iron deficiency response in rice (*Oryza sativa* L.). Plant Sci..

[134] Curie C., Briat J.-F. (2003). Iron transport and signaling in plants. Annu. Rev. Plant Biol..

[135] Cheng L., Wang F., Shou H., Huang F., Zheng L., He F., Li J., Zhao F.-J., Ueno D., Ma J.F. (2007). Mutation in nicotianamine aminotransferase stimulated the Fe(II) acquisition system and led to iron accumulation in rice. Plant Physiol..

[136] Zheng L., Cheng Z., Ai C., Jiang X., Bei X., Zheng Y., Raymond P., Welch R.M., Miller D.D., Lei X.G. (2010). Nicotianamine, a novel enhancer of rice iron bioavailability to humans. PLoS ONE.

[137] Lee S., Jeon U.S., Lee S.J., Kim Y.-K., Persson D.P., Husted S., Schjorring J.K., Kakei Y., Masuda H., Nishizawa N.K. (2009). Iron fortification of rice seeds through activation of the nicotianamine synthase gene. Proc. Natl. Acad. Sci. USA.

[138] Lee S., Kim Y.S., Jeon U.S., Kim Y.K., Schjoerring J.K., An G. (2012). Activation of rice *nicotianamine synthase 2* (*OsNAS2*) enhances iron availability for biofortification. Mol. Cells.

[139] Banakar R., Alvarez Fernandez A., Díaz-Benito P., Abadia J., Capell T., Christou P. (2017). Phytosiderophores determine thresholds for iron and zinc accumulation in biofortified rice endosperm while inhibiting the accumulation of cadmium. J. Exp. Bot..

[140] Singh S.P., Keller B., Gruissem W., Bhullar N.K. (2017). Rice *NICOTIANAMINE SYNTHASE 2* expression improves dietary iron and zinc levels in wheat. Theor. Appl. Genet..

[141] Masuda H., Usuda K., Kobayashi T., Ishimaru Y., Kakei Y., Takahashi M., Higuchi K., Nakanishi H., Mori S., Nishizawa N.K. (2009). Overexpression of the barley nicotianamine synthase gene *HvNAS1* increases iron and zinc concentrations in rice grains. Rice.

[142] Beasley J.T., Hart J.J., Tako E., Glahn R.P., Johnson A.A.T. (2019). Investigation of nicotianamine and 2′ deoxymugineic acid as enhancers of iron bioavailability in Caco-2 cells. Nutrients.

[143] Goto F., Yoshihara T., Shigemoto N., Toki S., Takaiwa F. (1999). Iron fortification of rice seed by the soybean ferritin gene. Nat. Biotechnol..

[144] Lucca P., Hurrell R., Potrykus I. (2001). Approaches to improving the bioavailability and level of iron in rice seeds. J. Sci. Food Agric..

[145] Vasconcelos M., Datta K., Oliva N., Khalekuzzaman M., Torrizo L., Krishnan S., Oliveira M., Goto F., Datta S.K. (2003). Enhanced iron and zinc accumulation in transgenic rice with the ferritin gene. Plant Sci..

[146] Oliva N., Chadha-Mohanty P., Poletti S., Abrigo E., Atienza G., Torrizo L., Garcia R., Dueñas C., Poncio M.A., Balindong J. (2014). Large-scale production and evaluation of marker-free indica rice IR64 expressing phytoferritin genes. Mol. Breed..

[147] Davila-Hicks P., Theil E.C., Lönnerdal B. (2004). Iron in ferritin or in salts (ferrous sulfate) is equally bioavailable in nonanemic women. Am. J. Clin. Nutr..

[148] Neal A.L., Geraki K., Borg S., Quinn P., Mosselmans J.F., Brinch-Pedersen H., Shewry P.R. (2013). Iron and zinc complexation in wild-type and ferritin-expressing wheat grain: Implications for mineral transport into developing grain. J. Biol. Inorg. Chem..

[149] Masuda H., Ishimaru Y., Aung M.S., Kobayashi T., Kakei Y., Takahashi M., Higuchi K., Nakanishi H., Nishizawa N.K. (2012). Iron biofortification in rice by the introduction of multiple genes involved in iron nutrition. Sci. Rep..

[150] Wu T.-Y., Gruissem W., Bhullar N.K. (2018). Targeting intracellular transport combined with efficient uptake and storage significantly increases grain iron and zinc levels in rice. Plant Biotechnol. J..

[151] Trijatmiko K.R., Dueñas C., Tsakirpaloglou N., Torrizo L., Arines F.M., Adeva C., Balindong J., Oliva N., Sapasap M.V., Borrero J. (2016). Biofortified indica rice attains iron and zinc nutrition dietary targets in the field. Sci. Rep..

[152] Saito K. (2004). Sulfur assimilatory metabolism. The long and smelling Road. Plant Physiol..

[153] Kopriva S. (2006). Regulation of sulfate assimilation in Arabidopsis and beyond. Ann. Bot..

[154] Fuentes-Lara L.O., Medrano-Macías J., Pérez-Labrada F., Rivas-Martínez E.N., García-Enciso E.L., González-Morales S., Juárez-Maldonado A., Rincón-Sánchez F., Benavides-Mendoza A. (2019). From elemental sulfur to hydrogen sulfide in agricultural soils and plants. Molecules.

[155] Pierzynski G.M., Sims J.T., Vance G.F. (2005). Soils and Environmental Quality.

[156] Ponnamperuma F.N. (1972). The Cchemistry of submerged soils. Adv. Agron..

[157] Achtnich C., Bak F., Conrad R. (1995). Competition for electron donors among nitrate reducers, ferric iron reducers, sulfate reducers, and methanogens in anoxic paddy soil. Biol. Fertil. Soils.

[158] Wind T., Conrad R. (1995). Sulfur compounds, potential turnover of sulfate and thiosulfate, and numbers of sulfate-reducing bacteria in planted and unplanted paddy soil. FEMS Microbiol. Ecol..

[159] Stubner S., Wind T., Conrad R. (1998). Sulfur oxidation in rice field soil: Activity, enumeration, isolation and characterization of thiosulfate-oxidizing bacteria. Syst. Appl. Microbiol..

[160] Hu Z., Hanekalus S., Cao Z., Schnug E., De Kok L.J., Schnug E. (2005). Chemical behavior of soil sulphur in the rhizosphere and its ecological effects. Proceedings of the 1st Sino-German Workshop on Aspects of Sulfur Nutrition of Plants; 23–27 May 2004 Shenyang, China.

[161] Takahashi H. (2010). Regulation of sulfate transport and assimilation in plants. International Review of Cell and Molecular Biology.

[162] Takahashi H., Kopriva S., Giordano M., Saito K., Hell R. (2011). Sulfur assimilation in photosynthetic organisms: Molecular functions and regulations of transporters and assimilatory enzymes. Annu. Rev. Plant Biol..

[163] Takahashi H., Buchner P., Yoshimoto N., Hawkesford M.J., Shiu S.H. (2012). Evolutionary relationships and functional diversity of plant sulfate transporters. Front. Plant Sci..

[164] Takahashi H., Watanabe-Takahashi A., Smith F.W., Blake-Kalff M., Hawkesford M.J., Saito K. (2000). The roles of three functional sulphate transporters involved in uptake and translocation of sulphate in Arabidopsis thaliana. Plant J..

[165] Shibagaki N., Rose A., McDermott J.P., Fujiwara T., Hayashi H., Yoneyama T., Davies J.P. (2002). Selenate-resistant mutants of Arabidopsis thaliana identify Sultr1;2, a sulfate transporter required for efficient transport of sulfate into roots. Plant J..

[166] Kumar S., Asif M.H., Chakrabarty D., Tripathi R.D., Trivedi P.K. (2011). Differential expression and alternative splicing of rice sulphate transporter family members regulate sulphur status during plant growth, development and stress conditions. Funct. Integr. Genom..

[167] Godwin R.M., Rae A.L., Carroll B.J., Smith F.W. (2003). Cloning and characterization of two genes encoding sulfate transporters from rice (*Oryza sativa* L.). Plant Soil.

[168] Smith F.W., Hawkesford M.J., Ealing P.M., Clarkson D.T., Vanden Berg P.J., Belcher A.R., Warrilow A.G.S. (1997). Regulation of expression of a cDNA from barley roots encoding a high affinity sulphate transporter. Plant J..

[169] Rae A.L., Smith F.W. (2002). Localisation of expression of a high-affinity sulfate transporter in barley roots. Planta.

[170] Vidmar J.J., Schjoerring J.K., Touraine B., Glass A.D.M. (1999). Regulation of the *hvst1* gene encoding a high-affinity sulfate transporter from Hordeum vulgare. Plant Mol. Biol..

[171] Bolchi A., Petrucco S., Tenca P.L., Foroni C., Ottonello S. (1999). Coordinate modulation of maize sulfate permease and ATP sulfurylase mRNAs in response to variations in sulfur nutritional status: Stereospecific down-regulation by L-cysteine. Plant Mol. Biol..

[172] Hopkins L., Parmar S., Bouranis D.L., Howarth J.R., Hawkesford M.J. (2004). Coordinated expression of sulfate uptake and components of the sulfate assimilatory pathway in maize. Plant Biol..

[173] Buchner P., Prosser I.M., Hawkesford M.J. (2004). Phylogeny and expression of paralogous and orthologous sulphate transporter genes in diploid and hexaploid wheats. Genome.

[174] Bell R.W., Jez J. (2008). Sulfur and the Production of Rice in Wetland and Dryland Ecosystems. Sulfur: A Missing Link between Soils, Crops, and Nutrition.

[175] Rausch T., Wachter A. (2005). Sulfur metabolism: A versatile platform for launching defence operations. Trends Plant Sci..

[176] Kataoka T., Hayashi N., Yamaya T., Takahashi H. (2004). Root-to-shoot transport of sulfate in Arabidopsis. Evidence for the role of SULTR3;5 as a component of low-affinity sulfate transport system in the root vasculature. Plant Physiol..

[177] Moura J.J.G., Bursakov S.A., Gavel O., Moura I. (2002). Sulfate activation. Encycl. Catal..

[178] Leustek T., Murillo M., Cervantes M. (1994). Cloning of a cDNA encoding ATP sulfurylase from *Arabidopsis thaliana* by functional expression in Saccharomyces cerevisiae. Plant Physiol..

[179] Setya A., Murillo M., Leustek T. (1996). Sulfate reduction in higher plants: Molecular evidence for a novel 5′-adenylylsulfate reductase. Proc. Natl. Acad. Sci. USA.

[180] Nakayama M., Akashi T., Hase T. (2000). Plant sulfite reductase: Molecular structure, catalytic function and interaction with ferredoxin. J. Inorg. Biochem..

[181] Brühl A., Haverkamp T., Gisselmann G., Schwenn J.D. (1996). A cDNA clone from *Arabidopsis thaliana* encoding plastidic gerredoxin: Sulfite reductase. Biochim. Biophys. Acta Protein Struct. Mol. Enzymol..

[182] Saito K., Miura N., Yamazaki M., Hirano H., Murakoshi I. (1992). Molecular cloning and bacterial expression of cDNA encoding a plant cysteine synthase. Proc. Natl. Acad. Sci. USA.

[183] Hell R., Bork C., Bogdanova N., Frolov I., Hauschild R. (1994). Isolation and characterization of two cDNAs encoding for compartment specific isoforms of *O*-acetylserine (thiol) lyase from Arabidopsis thaliana. FEBS Lett..

[184] Noji M., Inoue K., Kimura N., Gouda A., Saito K. (1998). Isoform-dependent differences in feedback regulation and subcellular localization of serine acetyltransferase involved in cysteine biosynthesis from *Arabidopsis thaliana*. J. Biol. Chem..

[185] Hell R., Wirtz M., Hell R., Dahl C., Knaff D.B., Leustek T. (2008). Metabolism of cysteine in plants and phototrophic bacteria. Sulfur Metabolism in Phototrophic Organisms.

[186] Lu Y. (2018). Assembly and transfer of iron–sulfur clusters in the plastid. Front. Plant Sci..

[187] Balk J., Pilon M. (2011). Ancient and essential: The assembly of iron-sulfur clusters in plants. Trends Plant Sci..

[188] Na G.N., Salt D.E. (2011). The role of sulfur assimilation and sulfur-containing compounds in trace element homeostasis in plants. Environ. Exp. Bot..

[189] Ravanel S., Gakiere B., Job D., Douce R. (1998). The specific features of methionine biosynthesis and metabolism in plants. Proc. Natl. Acad. Sci. USA.

[190] Ravanel S., Block M.A., Rippert P., Jabrin S., Curien G., Rébeillé F., Douce R. (2004). Methionine metabolism in plants: Chloroplasts are autonomous for de novo methionine synthesis and can import S-adenosylmethionine from the cytosol. J. Biol. Chem..

[191] Roje S. (2006). *S*-Adenosyl-L-methionine: Beyond the universal methyl group donor. Phytochemistry.

[192] Takahashi M., Terada Y., Nakai I., Nakanishi H., Yoshimura E., Mori S., Nishizawa N.K. (2003). Role of nicotianamine in the intracellular delivery of metals and plant reproductive development. Plant Cell.

[193] Marschner H. (1995). Functions of mineral nutrients: Macronutrients. Mineral Nutrition of Higher Plants.

[194] Noctor G., Mhamdi A., Chaouch S., Han Y., Neukermans J., Marquez-Garcia B., Queval G., Foyer C.H. (2012). Glutathione in plants: An integrated overview. Plant Cell Environ..

[195] Wang F., Chen F., Cai Y., Zhang G., Wu F. (2011). Modulation of exogenous glutathione in ultrastructure and photosynthetic performance against Cd stress in the two barley genotypes differing in Cd tolerance. Biol. Trace Elem. Res..

[196] Chen F., Wang F., Wu F., Mao W., Zhang G., Zhou M. (2010). Modulation of exogenous glutathione in antioxidant defense system against Cd stress in the two barley genotypes differing in Cd tolerance. Plant Physiol. Biochem..

[197] Yamazaki S., Ueda Y., Mukai A., Ochiai K., Matoh T. (2018). Rice phytochelatin synthases OsPCS1 and OsPCS2 make different contributions to cadmium and arsenic tolerance. Plant Direct.

[198] Hell R., Bergmann L. (1990). λ-Glutamylcysteine synthetase in higher plants: Catalytic properties and subcellular localization. Planta.

[199] Wang C.L., Oliver D.J. (1996). Cloning of the cDNA and genomic clones for glutathione synthetase from *Arabidopsis thaliana* and complementation of a *gsh2* mutant in fission yeast. Plant Mol. Biol..

[200] Cobbett C., Goldsbrough P. (2002). Phytochelatins and metallothioneins: Roles in heavy metal detoxification and homeostasis. Annu. Rev. Plant Biol..

[201] Vatamaniuk O.K., Mari S., Lu Y.-P., Rea P.A. (1999). AtPCS1, a phytochelatin synthase from Arabidopsis: Isolation and *in vitro* reconstitution. Proc. Natl. Acad. Sci. USA.

[202] Clemens S., Kim E.J., Neumann D., Schroeder J.I. (1999). Tolerance to toxic metals by a gene family of phytochelatin synthases from plants and yeast. EMBO J..

[203] Song W.Y., Mendoza-Cózatl D.G., Lee Y., Schroeder J.I., Ahn S.N., Lee H.S., Wicker T., Martinoia E. (2014). Phytochelatin-metal(loid) transport into vacuoles shows different substrate preferences in barley and Arabidopsis. Plant Cell Environ..

[204] Uraguchi S., Tanaka N., Hofmann C., Abiko K., Ohkama-Ohtsu N., Weber M., Kamiya T., Sone Y., Nakamura R., Takanezawa Y. (2017). Phytochelatin synthase has contrasting effects on cadmium and arsenic accumulation in rice grains. Plant Cell Physiol..

[205] Hasan M.K., Cheng Y., Kanwar M.K., Chu X.Y., Ahammed G.J., Qi Z.Y. (2017). Responses of plant proteins to heavy metal stress—A review. Front. Plant Sci..

[206] Hegelund J.N., Schiller M., Kichey T., Hansen T.H., Pedas P., Husted S., Schjoerring J.K. (2012). Barley metallothioneins: MT3 and MT4 are localized in the grain aleurone layer and show Differential zinc binding. Plant Physiol..

[207] Mekawy A.M.M., Assaha D.V.M., Munehiro R., Kohnishi E., Nagaoka T., Ueda A., Saneoka H. (2018). Characterization of type 3 metallothionein-like gene (*OsMT-3a*) from rice, revealed its ability to confer tolerance to salinity and heavy metal stresses. Environ. Exp. Bot..

[208] Nezhad R.M., Shahpiri A., Mirlohi A. (2013). Discrimination between two rice metallothionein isoforms belonging to type 1 and type 4 in metal-binding ability. Biotechnol. Appl. Biochem..

[209] Lane B., Kajioka R., Kennedy T. (1987). The wheat-germ E c protein is a zinc-containing metallothionein. Biochem. Cell Biol..

[210] Yu L.H., Umeda M., Liu J.Y., Zhao N.M., Uchimiya H. (1998). A novel MT gene of rice plants is strongly expressed in the node portion of the stem. Gene.

[211] Zhang H., Lv S., Xu H., Hou D., Li Y., Wang F. (2017). H_2_O_2_ is involved in the metallothionein-mediated rice tolerance to copper and cadmium toxicity. Int. J. Mol. Sci..

[212] Yang Z., Wu Y., Li Y., Ling H.-Q., Chu C. (2009). OsMT1a, a type 1 metallothionein, plays the pivotal role in zinc homeostasis and drought tolerance in rice. Plant Mol. Biol..

[213] Li Z.G., Cadenas E., Packer L. (2015). Analysis of some enzymes activities of hydrogen sulfide metabolism in plants. Methods in Enzymology.

[214] Li Z.G., Min X., Zhou Z.H. (2016). Hydrogen sulfide: A signal molecule in plant cross-adaptation. Front. Plant Sci..

[215] Cao M.J., Wang Z., Wirtz M., Hell R., Oliver D.J., Xiang C. (2013). Bin SULTR3;1 is a chloroplast-localized sulfate transporter in *Arabidopsis thaliana*. Plant J..

[216] Kataoka T., Watanabe-Takahashi A., Hayashi N., Ohnishi M., Mimura T., Buchner P., Hawkesford M.J., Yamaya T., Takahashi H. (2004). Vacuolar sulfate transporters are essential determinants controlling internal distribution of sulfate in Arabidopsis. Plant Cell.

[217] Gigolashvili T., Kopriva S. (2014). Transporters in plant sulfur metabolism. Front. Plant Sci..

[218] Herschbach C., Rennenberg H., Esser K., Lüttge U., Kadereit J.W., Beyschlag W. (2001). Significance of phloem-translocated organic sulfur compounds for the regulation of sulfur nutrition. Progress in Botany.

[219] Bourgis F., Roje S., Nuccio M.L., Fisher D.B., Tarczynski M.C., Li C., Herschbach C., Rennenberg H., Pimenta M.J., Shen T.L. (1999). *S*-methylmethionine plays a major role in phloem sulfur transport and is synthesized by a novel type of methyltransferase. Plant Cell.

[220] Kuzuhara Y., Isobe A., Awazuhara M., Fujiwara T., Hayashi H. (2000). Glutathione levels in phloem sap of rice plants under sulfur deficient conditions. Soil Sci. Plant Nutr..

[221] Cagnac O., Bourbouloux A., Chakrabarty D., Zhang M.Y., Delrot S. (2004). AtOPT6 transports glutathione derivatives and is induced by primisulfuron. Plant Physiol..

[222] Hayashi H., Chino M. (1985). Nitrate and other anions in the rice phloem sap. Plant Cell Physiol..

[223] Yoshimoto N., Inoue E., Saito K., Yamaya T., Takahashi H. (2003). Phloem-localizing sulfate transporter, Sultr1;3, mediates re-distribution of sulfur from source to sink organs in Arabidopsis. Plant Physiol..

[224] Wu Z., Zhang C., Dai C., Ge Y. (2015). Sufficient sulfur supply promotes seedling growth, alleviates oxidation stress, and regulates iron uptake and translocation in rice. Biol. Plant..

[225] Astolfi S., Cesco S., Zuchi S., Neumann G., Roemheld V. (2006). Sulfur starvation reduces phytosiderophores release by iron-deficient barley plants. Soil Sci. Plant Nutr..

[226] Zuchi S., Cesco S., Astolfi S. (2012). High S supply improves Fe accumulation in durum wheat plants grown under Fe limitation. Environ. Exp. Bot..

[227] Astolfi S., Zuchi S., Passera C., Cesco S. (2003). Does the sulfur assimilation pathway play a role in the response to Fe deficiency in maize (*Zea mays* L.) plants. J. Plant Nutr..

[228] Ciaffi M., Paolacci A.R., Celletti S., Catarcione G., Kopriva S., Astolfi S. (2013). Transcriptional and physiological changes in the S assimilation pathway due to single or combined S and Fe deprivation in durum wheat (*Triticum durum* L.) seedlings. J. Exp. Bot..

[229] Wu Z., Naveed S., Zhang C., Ge Y. (2020). Adequate supply of sulfur simultaneously enhances iron uptake and reduces cadmium accumulation in rice grown in hydroponic culture. Environ. Pollut..

[230] Wu C.Y.H., Lu J., Hu Z.Y. (2014). Influence of sulfur supply on the iron accumulation in rice plants. Commun. Soil Sci. Plant Anal..

[231] Kuwajima K., Kawai S. (1997). Relationship between sulfur metabolism and biosynthesis of phytosiderophores in barley roots. Plant Nutrition for Sustainable Food Production and Environment.

[232] Inoue H., Higuchi K., Takahashi M., Nakanishi H., Mori S., Nishizawa N.K. (2003). Three rice nicotianamine synthase genes, *OsNAS1*, *OsNAS2*, and *OsNAS3* are expressed in cells involved in long-distance transport of iron and differentially regulated by iron. Plant J..

[233] Higuchi K., Takahashi M., Nakanishi H., Kawasaki S., Nishizawa N.K., Mori S. (2001). Analysis of transgenic rice containing barley nicotianamine synthase gene. Soil Sci. Plant Nutr..

[234] Takahashi M., Nakanishi H., Kawasaki S., Nishizawa N.K., Mori S. (2001). Enhanced tolerance of rice to low iron availability in alkaline soils using barley nicotianamine aminotransferase genes. Nat. Biotechnol..

[235] Banakar R., Fernandez A.A., Zhu C., Abadia J., Capell T., Christou P. (2019). The ratio of phytosiderophores nicotianamine to deoxymugenic acid controls metal homeostasis in rice. Planta.

[236] Díaz-Benito P., Banakar R., Rodríguez-Menéndez S., Capell T., Pereiro R., Christou P., Abadía J., Fernández B., Álvarez-Fernández A. (2018). Iron and zinc in the embryo and endosperm of rice (*Oryza sativa* L.) seeds in contrasting 2′-deoxymugineic acid/nicotianamine scenarios. Front. Plant Sci..

[237] Haneklaus S., Bloem E., Schnug E., Jez J. (2008). History of Sulfur Deficiency in Crops. Sulfur: A Missing Link between Soils, Crops, and Nutrition.

[238] Lucheta A.R., Lambais M.R. (2012). Sulfur in agriculture. Rev. Bras. Ciência Solo.

[239] Zhao F., Tausz M., De Kok L.J., Hell R., Dahl C., Knaff D., Leustek T. (2008). Role of sulfur for plant production in agricultural and natural ecosystems. Sulfur Metabolism in Phototrophic Organisms.

[240] Yoshimoto N., Inoue E., Watanabe-Takahashi A., Saito K., Takahashi H. (2007). Posttranscriptional regulation of high-affinity sulfate transporters in arabidopsis by sulfur nutrition. Plant Physiol..

[241] Chen J., Wu F.H., Shang Y.T., Wang W.H., Hu W.J., Simon M., Liu X., Shangguan Z.P., Zheng H.L. (2015). Hydrogen sulphide improves adaptation of *Zea mays* seedlings to iron deficiency. J. Exp. Bot..

[242] Zhang X., Zhang Y., Zhang L., Zhao H., Li H. (2017). Hydrogen sulphide improves iron homeostasis in wheat under iron-deficiency. J. Plant Sci..

[243] Lisjak M., Teklic T., Wilson I.D., Whiteman M., Hancock J.T. (2013). Hydrogen sulfide: Environmental factor or signalling molecule?. Plant. Cell Environ..

[244] Chen J., Shangguan Z.P., Zheng H.L. (2016). The function of hydrogen sulphide in iron availability: Sulfur nutrient or signaling molecule?. Plant Signal. Behav..

[245] Ogo Y., Itai R.N., Kobayashi T., Aung M.S., Nakanishi H., Nishizawa N.K. (2011). OsIRO2 is responsible for iron utilization in rice and improves growth and yield in calcareous soil. Plant Mol. Biol..

[246] Vámos R. (1958). H_2_S, the cause of the bruzone (Akiochi) disease of rice. Soil Sci. Plant Nutr..

[247] Dobermann A., Fairhurst T. (2000). Rice: Nutrient Disorders & Nutrient Management.

[248] Armstrong J., Armstrong W. (2005). Rice: Sulfide-induced barriers to root radial oxygen loss, Fe^2+^ and water uptake, and lateral root emergence. Ann. Bot..

[249] Groth D., Lee F., Smith C.W., Dilday R.H. (2002). Rice diseases. Rice: Origin, History, Technology, and Production.

[250] Joshi M.M. (1975). Hydrogen sulfide: Effects on the physiology of rice plants and relation to straighthead disease. Phytopathology.

[251] Yang J.X., Liu Y., Ye Z.H. (2012). Root-induced changes of pH, Eh, Fe(II) and fractions of Pb and Zn in rhizosphere soils of four wetland plants with different radial oxygen losses. Pedosphere.

[252] LaFond-Hudson S., Johnson N.W., Pastor J., Dewey B. (2018). Iron sulfide formation on root surfaces controlled by the life cycle of wild rice (*Zizania palustris*). Biogeochemistry.

[253] Schmidt H., Eickhorst T., Tippkötter R. (2011). Monitoring of root growth and redox conditions in paddy soil rhizotrons by redox electrodes and image analysis. Plant Soil.

[254] Yang J., Liu Z., Wan X., Zheng G., Yang J., Zhang H., Guo L., Wang X., Zhou X., Guo Q. (2016). Interaction between sulfur and lead in toxicity, iron plaque formation and lead accumulation in rice plant. Ecotoxicol. Environ. Saf..

[255] Zhang X., Zhang F., Mao D. (1999). Effect of iron plaque outside roots on nutrient uptake by rice (*Oryza sativa* L.): Phosphorus uptake. Plant Soil.

[256] Zhou X.-B., Shi W.-M. (2007). Effect of root surface iron plaque on Se translocation and uptake by Fe-deficient rice. Pedosphere.

[257] Zhang X., Zhang F., Mao D. (1998). Effect of iron plaque outside roots on nutrient uptake by rice (*Oryza sativa* L.). Zinc uptake by Fe-deficient rice. Plant Soil.

[258] Nozoye T., Itai R.N., Nagasaka S., Takahashi M., Nakanishi H., Mori S., Nishizawa N.K. (2004). Diurnal changes in the expression of genes that participate im phytosiderophore synthesis in rice. Soil Sci. Plant Nutr..

[259] Murakami T., Ise K., Hayakawa M., Kamei S., Takagi S. (1989). Stabilities of Metal Complexes of Mugineic Acids and Their Specific Affinities for Iron(III). Chem. Lett..

[260] Qin Y., Song F., Ai Z., Zhang P., Zhang L. (2015). Protocatechuic acid promoted alachlor degradation in Fe(III)/H_2_O_2_ Fenton system. Environ. Sci. Technol..

[261] Freney J.R., Jacq V.A., Baldensperger J.F. (1982). The significance of the biological sulfur cycle in rice production. Microbiology of Tropical Soils and Plant Productivity.

[262] Attanandana T., Vacharotayan S. (1986). Acid sulfate soils: Their characteristics, genesis, amelioration and utilization. Southeast Asian Stud..

[263] Lee S., Persson D.P., Hansen T.H., Husted S., Schjoerring J.K., Kim Y.S., Jeon U.S., Kim Y.K., Kakei Y., Masuda H. (2011). Bio-available zinc in rice seeds is increased by activation tagging of *nicotianamine synthase*. Plant Biotechnol. J..

[264] Broadley M., Brown P., Cakmak I., Rengel Z., Zhao F., Marschner P. (2011). Function of Nutrients: Micronutrients. Marschner’s Mineral Nutrition of Higher Plants.

[265] Lin Y.F., Aarts M.G.M. (2012). The molecular mechanism of zinc and cadmium stress response in plants. Cell. Mol. Life Sci..

[266] Darbani B., Noeparvar S., Borg S. (2015). Deciphering mineral homeostasis in barley seed transfer cells at transcriptional level. PLoS ONE.

[267] Uraguchi S., Fujiwara T. (2012). Cadmium transport and tolerance in rice: Perspectives for reducing grain cadmium accumulation. Rice.

[268] Nakanishi H., Ogawa I., Ishimaru Y., Mori S., Nishizawa N.K. (2006). Iron deficiency enhances cadmium uptake and translocation mediated by the Fe^2+^ transporters OsIRT1 and OsIRT2 in rice. Soil Sci. Plant Nutr..

[269] Lee S., An G. (2009). Over-expression of *OsIRT1* leads to increased iron and zinc accumulations in rice. Plant Cell Environ..

[270] Tauris B., Borg S., Gregersen P.L., Holm P.B. (2009). A roadmap for zinc trafficking in the developing barley grain based on laser capture microdissection and gene expression profiling. J. Exp. Bot..

